# The miRNA-targeted transcriptome of porcine alveolar macrophages upon infection with Porcine Reproductive and Respiratory Syndrome Virus

**DOI:** 10.1038/s41598-019-39220-3

**Published:** 2019-02-28

**Authors:** Sophie Dhorne-Pollet, Elisa Crisci, Nuria Mach, Patricia Renson, Florence Jaffrézic, Guillemette Marot, Tatiana Maroilley, Marco Moroldo, Jérôme Lecardonnel, Fany Blanc, Nicolas Bertho, Olivier Bourry, Elisabetta Giuffra

**Affiliations:** 1 GABI, INRA, AgroParisTech, Université Paris Saclay, Jouy-en-Josas, 78350 France; 2 ANSES, Unité Virologie Immunologie Porcines, Ploufragan, 22440 France; 3 EA 2694 Biostatistiques, Université de Lille, Inria Lille Nord Europe, MODAL, Villeneuve d’Ascq, 59650 France; 4 Virologie et Immunologie Moléculaire, Institut National de la Recherche Agronomique, Université Paris-Saclay, Jouy-en-Josas, France; 5 Present Address: Department of Population Health and Pathobiology, College of Veterinary Medicine, North Carolina State University, Raleigh, NC United States; 6 Present Address: Departments of Medical Genetics and Biochemistry & Molecular Biology, Alberta Children’s Hospital Research Institute (ACHRI), Cumming School of Medicine, University of Calgary, Calgary, Canada; 7 Present Address: PIPAE, BIOEPAR, INRA, ONIRIS, Nantes Atlantic National College of Veterinary Medicine, Nantes, 44307 France

## Abstract

Host miRNAs are known to modulate the cell response to virus infections. We characterized the miRNA-targeted transcriptome of porcine alveolar macrophages (PAMs) at early times after infection with a subtype 1.1 strain of PRRSV (Porcine Reproductive and Respiratory Syndrome Virus). We performed the immunoprecipitation of RISC (RNA-induced Silencing Complex) followed by microarray analysis of the RISC-bound miRNA targets (RIP-Chip) to evaluate the relative enrichment or depletion of expressed genes in RISC. The miRNA-mediated regulation occurred early after PRRSV infection and decreased fast (1,241 and 141 RISC-bound genes at 7 h and 10 h post-infection, respectively); it affected several cell functions with evidence of miRNA buffering of upregulated interferon-related genes. Eight miRNAs were highly enriched in RISC of both control and infected cells with no evidence of differential expression. Although miR-335-5p was the miRNA with most predicted targets among enriched RISC-bound genes, no effects on surface markers, cytokine expression and PRRSV replication were detected upon miR-335-5p mimics of primary PAMs. Our results do not point to specific miRNA-driven mechanisms regulating the early response to infection with this PRRSV 1.1 strain and indicate that the miRNome expressed by steady-state PAMs reacts promptly to counterbalance PRRSV infection by a pervasive modulation of host functions.

## Introduction

The Porcine Reproductive and Respiratory Syndrome Virus (PRRSV) emerged in the early 1990s and since then represents a major problem to the swine industry worldwide^1,2^. It is a member of the *Arteriviridae* family (order *Nidovirales*) of enveloped viruses with positive-sense single-stranded RNA genomes with tropism for cells of the monocyte/macrophage lineage in lungs, lymphoid tissues and placenta^3^. Current PRRSV strains originate from two genetically and antigenically distinct viruses^4^ (European PRRSV I and American PRRSV II) and circulate globally^2,5,6^. As all RNA viruses, PRRSV is genetically unstable resulting in considerable genetic and virulence differences among PRRSV isolates and within genotypes^7,8^. Several studies have been published on all aspects of PRRSV in the last decades, with recent reviews on mechanisms of PRRSV pathogenesis and interaction with the host immune system^9^ and on recent technologies with potential to transform anti-PRRSV strategies^10^. Remarkably, the deletion by gene editing of a specific domain of the main cell receptor CD163 has allowed obtaining pigs which are resistant to main strains of PRRSV^11,12^.

MiRNAs are endogenous, non-protein coding small RNAs that modulate global gene expression in eukaryotic cells at the post-transcriptional level and are highly conserved across species^13–15^. Most miRNAs are initially transcribed by RNA polymerase II as part of a long pri-miRNA precursor that is processed by Drosha, in the nucleus, and Dicer, in the cytoplasm, to generate miRNA duplex intermediates. One strand of this duplex is then loaded into the RNA-induced silencing complex (RISC), which includes at least one of the four human Argonaute (Ago) proteins (Ago1 to Ago4), where it functions as a guide RNA to direct the RISC to fully or partially complementary mRNA targets. Loading into RISC leads to the release of the passenger strand into the cytoplasm, where it is degraded by exonucleases^16,17^. Commonly accepted principles of cooperativity (i.e. the targeting of a single mRNA by several miRNAs)^18^ and multiplicity (i.e. a single miRNA can target more than one gene)^19^ of miRNA function imply that individual mRNAs can be targeted by several miRNAs whereas a single miRNA may concomitantly regulate a subset of different genes. The interplay between cooperativity and multiplicity enables a single miRNA to fine tune protein biosynthesis from thousands of genes and to form complex regulatory networks within the cell^20,21^. As a result, miRNAs are critically involved in virtually all cellular processes, with evidence for a substantial degree of redundancy among miRNAs to maintain cellular homeostasis^22^.

Host miRNAs (and, for some viruses, viral miRNAs) modulate viral pathogenesis and host response by targeting viral and/or host transcripts^23^. Studies on RNA viruses have shown that host miRNAs can affect RNA virus replication and pathogenesis through direct binding to the RNA virus genome or through virus-mediated changes in the host transcriptome^24^. Such studies pave the bases for the design of antiviral therapeutic strategies and innovative vaccines, e.g. viruses can be engineered to become targets of a chosen host miRNA to restrict their tropism and reduce their pathogenicity^25^. PRRSV has been extensively investigated on these aspects, with several individual miRNAs reported to play important roles in PRRSV infection and replication, and in modulating host antiviral responses (reviewed by)^26^. However, these studies have made use of different cell systems, PRRSV strains, infection conditions and choice of time points for *in vitro* kinetics, making it difficult to infer unique or common patterns of miRNA host response between studies. Moreover, most of these studies focused on characterization and validation of individual, or few, miRNA-mRNA interactions.

Here, we aimed at identifying the whole set of host genes undergoing miRNA-mediated post-transcriptional regulation (i.e. the miRNA-targeted transcriptome) in the main target cells of PRRSV (porcine alveolar macrophages) during the early infection phase, when miRNAs may determine phenotypes not yet overruled by the cell immune responses or by cell death and other indirect effects. To this issue we carried out the immunoprecipitation of RISC followed by microarray analysis of the RISC-bound miRNA targets (RIP-Chip), as this high-throughput biochemical assay allows the genome-wide identification of genes targeted by cellular miRNAs in an unbiased and physiologically relevant manner^27,28^.

## Results

### Experimental design

**Figure 1:**
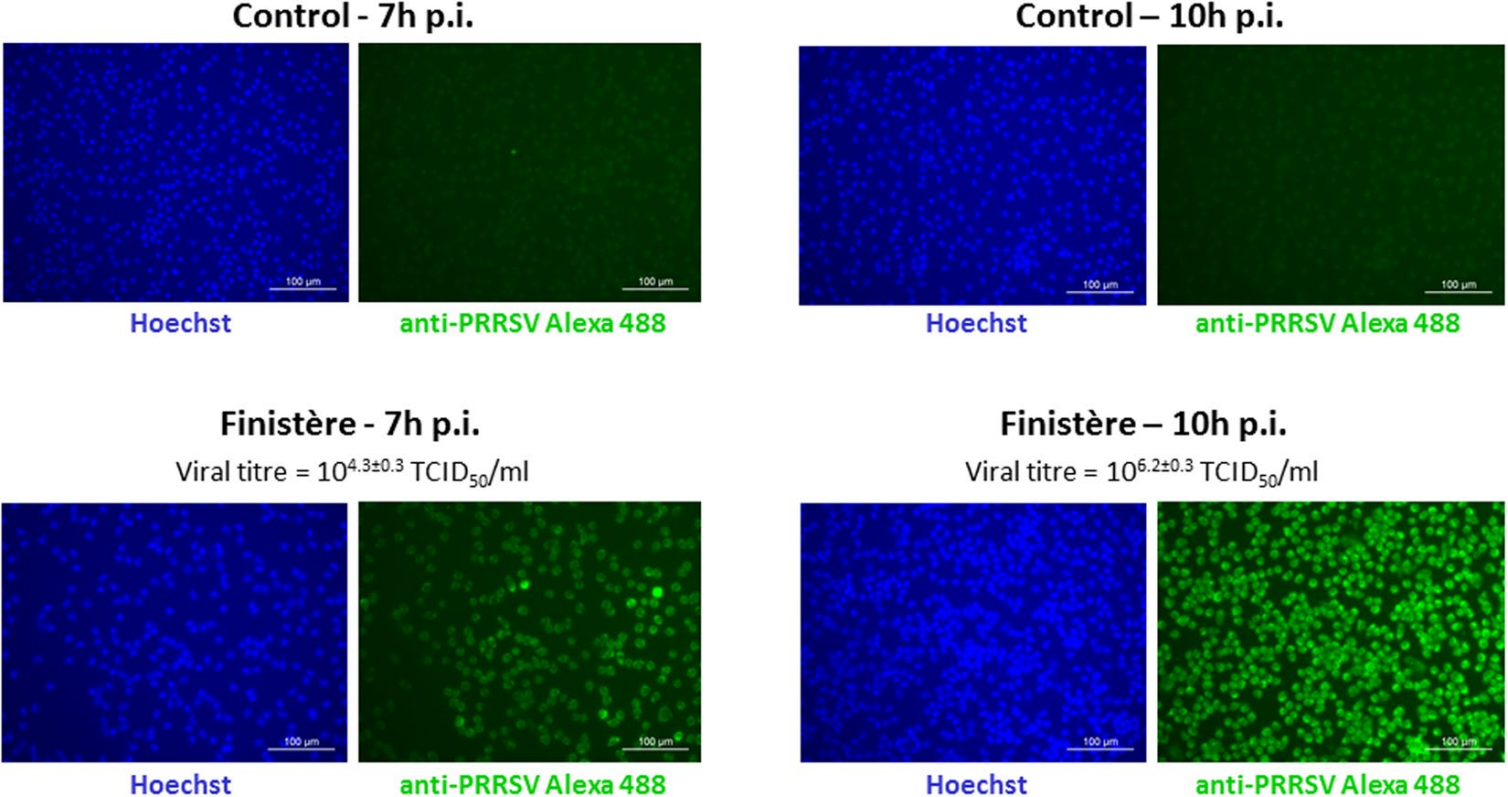
PRRSV immunofluorescence staining of PAMs infected with the Finistère strain at 7 h and 10 h post-infection (at MOI = 2) and controls. PRRSV indirect staining was performed with anti-PRRSV N protein antibody and anti-IgG Alexa 488-conjugated antibody (green). The nuclei were stained with Hoechst (blue). Magnification: 200X. Images are representative of two biological replicates with three technical replicates for each experimental condition. Viral titers are means ± standard deviations of two biological replicates with two technical replicates for each experimental condition.

The *in vitro* experimental infection with an European, low virulent PRRSV-1.1 strain (“Finistère”)^29^ was performed on PAMs isolated by bronchoalveolar lavages from four specific-pathogen-free piglets, with multiplicity of infection (MOI) of 2. Approximately 10^8^ PAMs were used for each experimental condition to ensure equal RNA input amounts for each microarray hybridization (50 ng and 10 ng of whole cell and RISC-bound RNA, respectively). In order to span only the first and early second replicative viral phase, the timing of cell harvesting was set at 7 h and 10 h post-infection (p.i.). As control, mock-infected PAMs of each individual were collected at the same times (7 h and 10 h p.i.) to account for modulation of the PAMs’ transcriptome occurring during cell culture independently on PRRSV infection. The average viral titres in culture media were 10^4.3±0.3^ and 10^6.2±0.3^ TCID_50_/ml at 7 h pi and 10 h pi, respectively, and no cytopathic effect was observed at both times. Immunostaining for PRRSV (N protein) indicated that at 7 h pi 100% of cells were infected by PRRSV, followed by more intense staining at 10 h p.i. (Fig. 1).

### Different dynamics and limited overlap of the whole cell and RISC-bound transcriptomes

**Figure 2:**
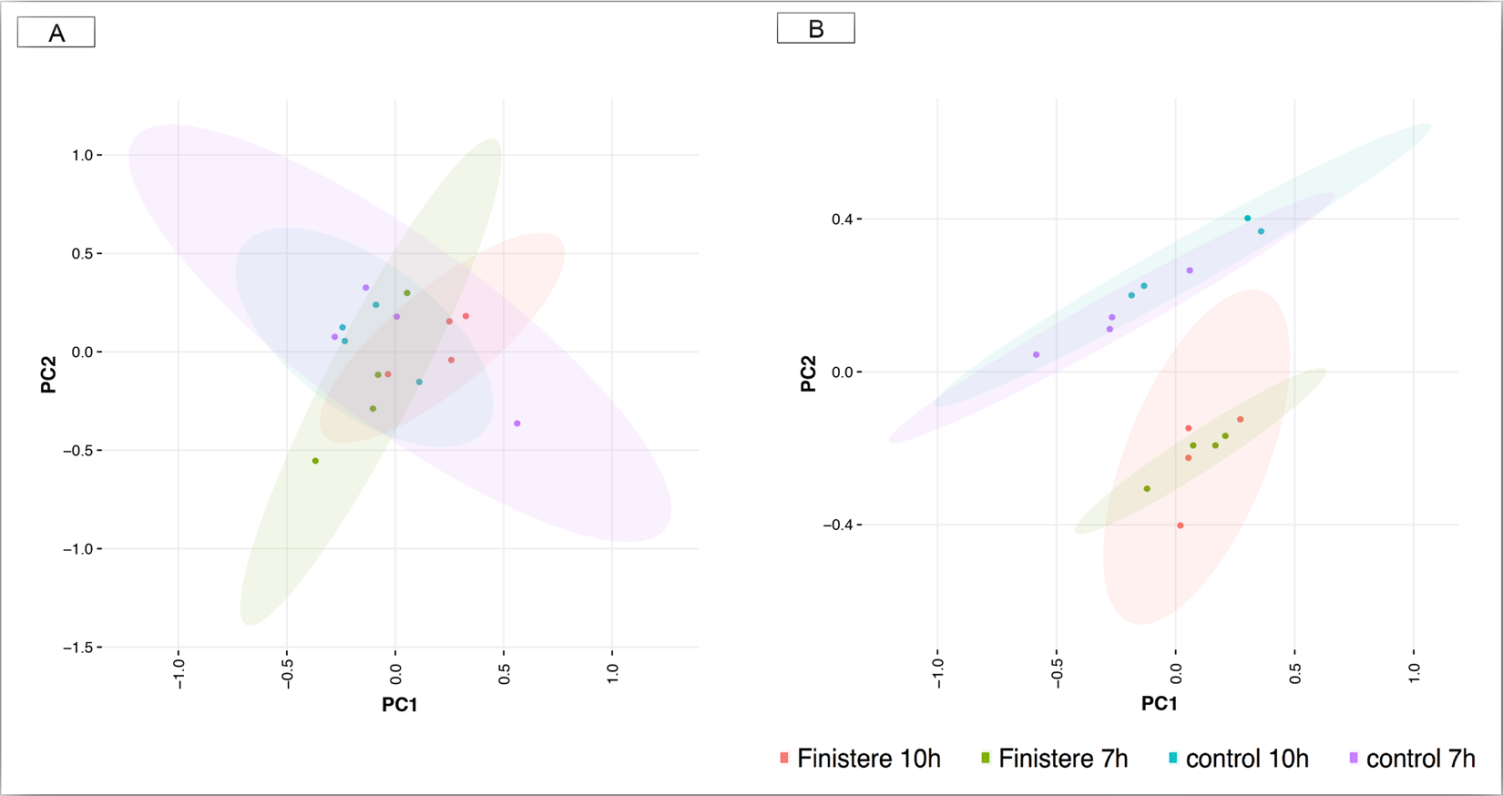
PCA of expression profiles in the whole cell and RISC. (**A**) PCA of transcriptome data in the whole cell at 7 h and 10 h post-infection and controls. (**B**) PCA of transcriptome data in RISC compartment at 7 h and 10 h post-infection and controls. The first axis accounted for 50.29% of the total variance, and the two axes accounted for 68.05% of the total variance. The 95% confidence ellipses were drawn to detect possible outliers in the study.

We first carried out an exploratory multivariate analysis on the normalized gene expression values in the whole cell and RISC compartments. The principal component analysis (PCA) showed a large overlap of the genes expressed in the whole cell compartment. These samples clustered together regardless of whether they were infected or control, or whether they were analysed at 7 or 10 h p.i. (Fig. 2A). Conversely, the PCA of RISC-bound genes allowed a better distinction between infected and control samples at both time points (Fig. 2B), indicating a clear effect of PRRSV infection on RISC bound genes that was only slightly influenced by the heterogeneity within conditions and between samples. The first principal component accounted for 50.3% of the total variance, and the first two components accounted for 68.1% of the total variance (Fig. 2B). Moreover, in order to exclude experimental biases in the efficiency of RISC immunoprecipitation among groups of samples, we performed Western Blot analysis. This confirmed that the immunoprecipitation efficiency was comparable between infected and non-infected samples at both times p.i. (Supplementary Fig. S1).

Then we focused on the identification of genes whose expression was significantly altered at 7 h or 10 h post-infection relative to control. For large genes with multiple probe sets, at least one of the probe sets needed to be significant (adjusted p-value < 0.05) after Benjamin-Hochberg correction (Supplementary Tables S1 and S2). The application of this threshold led to the identification of 170 differentially expressed genes (DEGs) at 7 h p.i. in the whole cell, of which 101 were upregulated and 69 were downregulated relative to control. At 10 h p.i. the number of DEGs increased (n = 924) and most of them (n = 551) were downregulated compared to control (Table 1). A similar pattern of downregulation following the onset of host response has been previously observed in PAMs following infection with a similar PRRSV strain^30,31^.

**Table 1:** Number of genes identified as differentially expressed (DEG) in the whole cell and significantly depleted or enriched in the RISC compartment of PAMs upon PRRSV infection.

		Whole cell			RISC-bound		
		DEG-upreg.	DEG-downreg.	Total DEGs	RISC-enriched	RISC-depleted	Total RISC-bound
7 h p.i.	No. genes	101	69	170	325	916	1241
	FC	2.30 ± 0.7	0.56 ± 0.06		2.32 ± 0.35	0.38 ± 0.11	
10 h p.i.	No. genes	373	551	924	6	135	141
	FC	2.25 ± 1.37	0.58 ± 0.07		2.38 ± 0.62	0.41 ± 0.12	

FC: fold change value. Significant genes presented a FDR-adjusted p-values smaller than 0.05.

An opposite pattern was found in the RISC compartment. By applying the anota algorithm, which uses the analysis of partial variance to weight the contribution of whole mRNA expression to the observed mRNA changes in RISC levels^32^, a total of 1,241 statistically significant RISC-bound genes were found at 7 h p.i. while only 141 genes were found at 10 h p.i.. At 7 h p.i. only 325 genes displayed positive fold changes (FC) >1 and thus are referred to here as “enriched RISC-bound” genes, while most genes (n = 916) became depleted (FC < 1). RISC-bound depleted genes also predominated at 10 h p.i. (135 out of 141 statistically significant genes) (Tables 1, S3 and S4). Both the prevalence of depleted genes and the average FC values found in the RISC compartment are in line with the results of previous studies in other host-virus systems^33,34^.

We found limited overlapping between the whole cell and RISC-bound transcriptomes upon PRRSV infection. Only 36 and 62 genes were both DEGs in the whole cell and statistically enriched or depleted in RISC at 7 h and 10 h p.i., respectively. The majority of enriched RISC-bound genes showed a trend to downregulation in the whole cell upon infection, which would be consistent with a reduction of transcriptional levels mediated by miRNAs. Specifically, 244 out of 325 genes at 7 h p.i. and all the six genes at 10 h p.i. had FC values below 0.9 in whole cell analyses.

Finally, we performed qPCR on a panel of 7 genes found as DEGs in the whole cell at 7 h and/or 10 h p.i. (*IFITM1*, interferon induced transmembrane protein 1; *GBP1*, guanylate binding protein 1; *ISG15*, ISG15 ubiquitin-like modifier; *HMGB1*, high mobility group box 1; *OAS2*, 2′-5′-oligoadenylate synthetase 2; *STAT1* signal transducer and activator of transcription 1; and *IFNB1*, interferon beta 1) and one of the transcription factors found enriched in RISC at 7 h p.i. (FOXO4, forkhead box O4). We used an independent panel of PAMs that were infected *in vitro* and processed for RISC immunoprecipitation in the same conditions as the first (RIP-Chip) experiment. Values and direction of gene expression changes in both the whole cell and RISC compartments were globally consistent with the microarray data (Table 2).

**Table 2:** Microarray (RIP-Chip experiment) and qPCR data of the expression of 8 genes in the whole cell and RISC compartments at 7 h and 10 h p.i.

Gene_Condition	Whole cell		RISC	
	qPCR	array	qPCR	array
*IFITM1*_7 h p.i.	1.89	4.46	1,68	n.s.
*IFITM1*_10 h p.i.	1.45	5.16	2.66	n.s.
*GBP1*_7 h p.i.	1.84	1.87	1.47	2.45
*GBP1*_10 h p.i.	2.59	2.17	2.45	n.s.
*ISG15*_7 h p.i.	2.22	3.28	2.14	5.33
*ISG15*_10 h p.i.	1.93	5.64	0.50	n.s.
*HMGB1*_7 h p.i.	1.27	n.s.	0.78	0.31
*HMGB1*_10 h p.i.	0.78	0.51	1.79	1.42
*OAS2*_7 h p.i.	1.75	4.78	1.73	n.s.
*OAS2*_10 h p.i.	1.45	4.14	2.31	5.18
*STAT1*_7 h p.i.	1.55	2.11	1.07	1.68
*STAT1*_10 h p.i.	1.20	n.s.	1.93	n.s.
*IFNB1*_7 h p.i.	385	n.s.	105	n.s.
*IFNB1*_10 h p.i.	11,765	4.29	2,535	n.s.
*FOXO4*_7 h p.i.	0.88	n.s.	0.91	1.71
*FOXO4*_10 h p.i.	0.68	n.s.	0.96	n.s.

The RNAs for the qPCRs were obtained from PAMs from a second independent panel of 4 pigs (see Materials and Methods). The qPCR values were normalized vs. the geometric mean of *GAPDH* and *B-Actin* and calibrated vs. non-infected controls. All data are expressed as linear fold change values; “n.s.”: non significant after RIP-Chip analysis. The two-sided Spearman’s Rho test of the correlation of qPCR and microarray data was statistically significant, with r_s_ = 0.76 and p (2-tailed) <0.001.

### Functional analysis of the whole cell and RISC compartments

#### Whole cell

**Figure 3:**
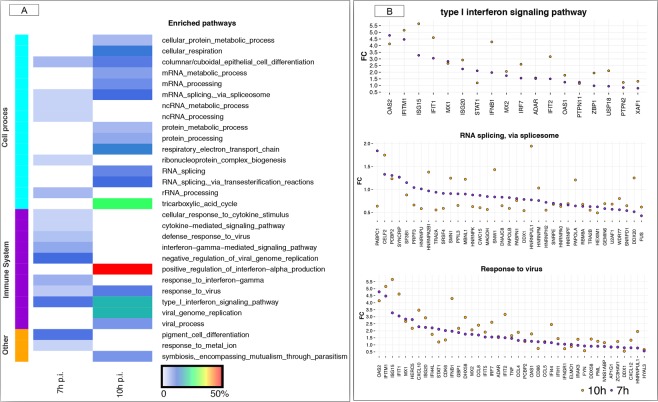
A functional map of DEGs in the whole cell. (**A**) The heatmap shows the top categories of enriched GO biological pathways at 7 h p.i. and 10 h p.i., respectively. The cell colours represent the % of DEGs associated with the various pathways. The lateral left colour represents the main biological function associated to each pathway. (**B**) Fold change representation of genes involved in the three commonly enriched pathways between 7 h and 10 h p.i. in the whole cell. In all plots, violet dots represent 7 h p.i. and orange dots represents 10 h p.i., respectively. Only those genes that were DEG either at 7 h or 10 h p.i. are plotted within the pathway.

As expected, the type I interferon signalling and response to virus were significantly enriched at both time points (Fig. 3A), with involved genes over-expressed in infected cells relative to control and some of them reaching FC values around 5 (Fig. 3B). The 170 DEGs at 7 h p.i. were mainly associated with the type I interferon signalling pathway, interferon gamma mediated signalling pathways and negative regulation of viral genome; the 924 DEGs at 10 h p.i were mainly associated to the positive regulation of interferon alpha production, followed by tricarboxylic acid (TCA) cycle, type I interferon signalling pathway and viral genome replication (Fig. 3A). The categories reflecting RNA metabolism and splicing via spliceosome processes were highly enriched, with most of the genes associated to RNA splicing downregulated in the course of infection compared to control (Fig. 3B). The full list of significantly enriched pathways at false discovery rate (FDR) <0.05 in the whole cell at 7 h p.i. and 10 h p.i. is given in Supplementary Tables S5 and S6, respectively. This pattern is largely consistent with previous studies using similar low virulent PRRSV-1.1 strains and early infection times^30,31^.

We also identified 16 transcription factors (TFs) out of the 170 DEGs (about 9%) in the whole cell at 7 h p.i. including *STAT1*, a main mediator of IFN-activated signalling (Supplementary Fig. S2A). At 10 h p.i., the relative proportion of TFs slightly decreased (56 out of 924; about 6%) (Supplementary Fig. S2B); among them, *HMGB1* is necessary for the host innate recognition of viruses nucleic acids^35^ and was reported by a meta-analysis study as the most significant TF of the pig specific response to PRRSV^36^. At 10 h p.i., *STAT1* was identified as the main upstream candidate TF (discovered using iRegulon) within the set of DEGs, controlling the expression changes of 98 genes out of 924 DEGs.

#### RISC

**Figure 4:**
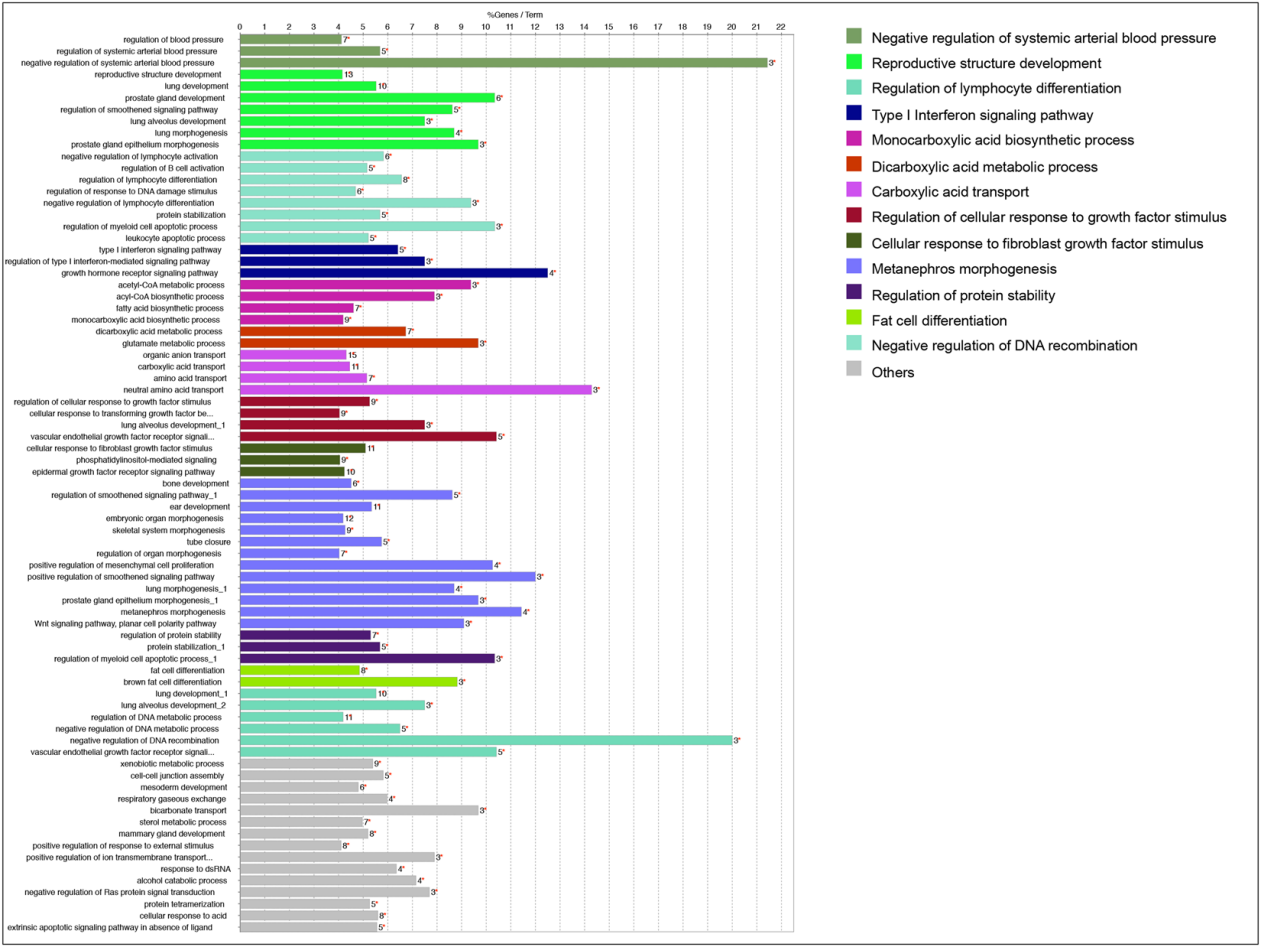
A functional map of enriched genes in the RISC compartment at 7 h p.i. The barplot shows the top categories of GO biological processes (which are coloured differently) associated with significantly enriched genes following infection. The bar length represent the percentage of enriched genes associated with the various terms. The number of genes in each term is also indicated.

Differently from the pattern observed in the whole cell, the 325 enriched-RISC bound genes at 7 h p.i. were involved with a large and diversified number of cellular pathways (Fig. 4). Immune-related pathways were very diversified themselves. The negative regulation of lymphocyte differentiation was significantly altered, with the occurrence of enriched genes within the GO term superior to 9%. The type I interferon-signalling pathway was represented by five genes (7% of genes associated with the GO term).

We identified 34 TFs out of the 325 enriched RISC-bound genes 7 h p.i. (Supplementary Fig. S3), thus in a similar proportion (about 10%) compared with the whole cell. The 34 TFs enriched in the RISC at 7 h p.i. included *STAT1*, which in combination with *FOXO4* and *GATA5* (GATA binding protein 5) potentially bind to 55% of the enriched RISC-bound genes (Supplementary Fig. S4).

### Patterns of miRNA targeting of RISC-bound genes

For most of the RISC-bound genes the annotation of 3′-UTRs is to date incomplete in the pig genome. However, when predicting miRNA targets in 3′ UTRs, analysis of conservation at the interspecific level can provide evidence that a predicted miRNA target is functional because it is being selected for, and indeed inter-specific conservation remains one of the main commonly used features for miRNA target prediction tools^37^. Thus, we first assembled a comprehensive list of all target genes experimentally validated in humans using the multiMiR R-package (see Materials and Methods). Then, we restricted the analysis to conserved miRNAs with expected biological function in primary alveolar macrophages; specifically, we considered only miRNAs found expressed in bovine alveolar macrophages^38^. Although relying on inter-specific conservation implies an underestimation of an organism’s and cell/tissue-specific repertoire of miRNAs^39^, the bovine and pig species are phylogenetically close and we considered it unlikely that new miRNAs evolved and acquired central roles in main regulatory circuits of innate immunity only in pigs^40^.

**Figure 5:**
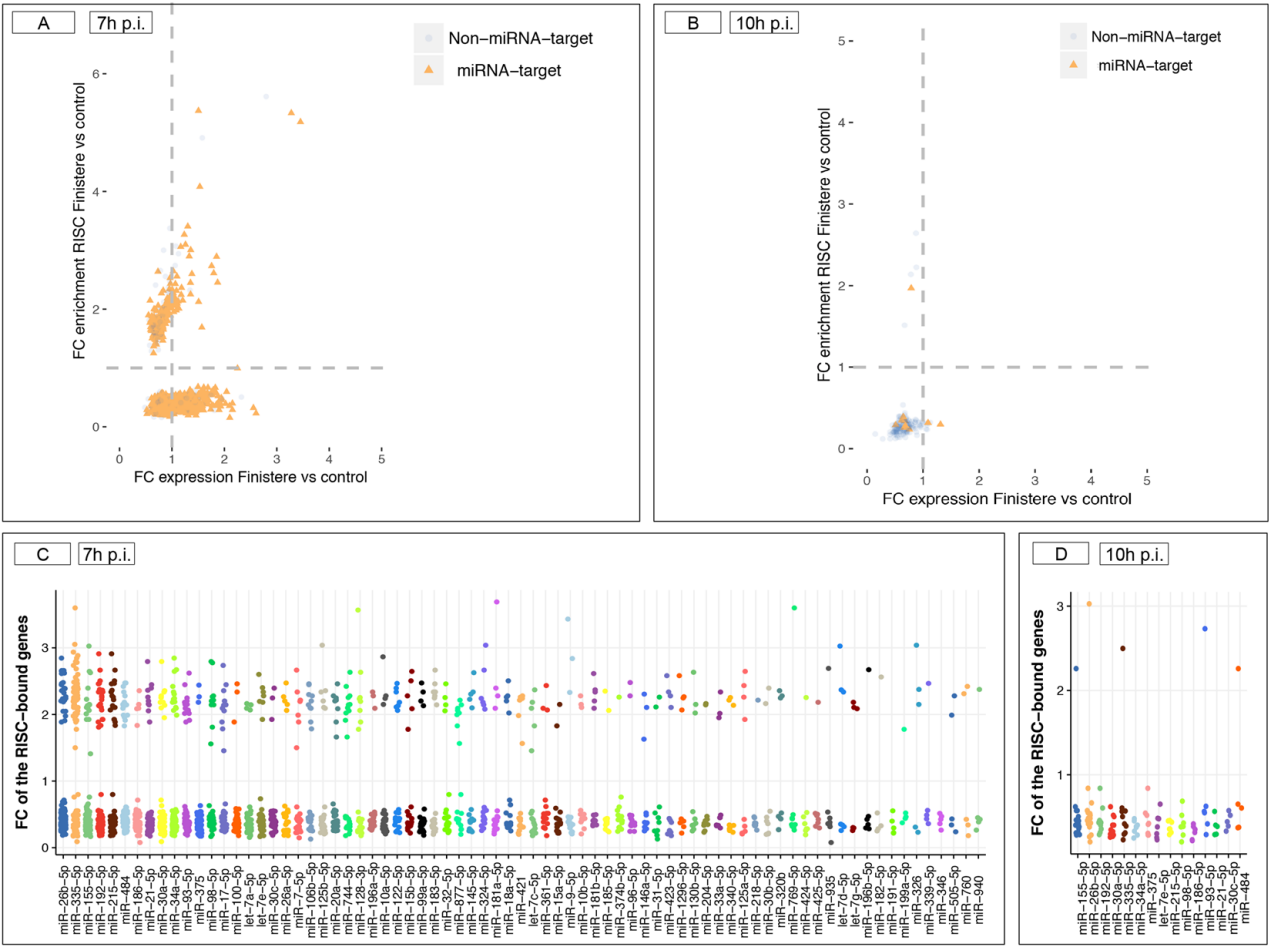
Putative targets of host miRNAs among enriched/ depleted RISC-bound genes. (**A**) Representation of putative target genes found within the 1,241 RISC-bound genes at 7 h p.i. The scatter plot depicts changes in mRNA levels in the whole cell (*x* axis) and in the RISC compartment (*y* axis). Orange dots indicate genes that are predicted target of at least one miRNA. (**B**) Same representation as (**A**) for the 141 RISC-bound genes statistically significant in RISC at 10 h p.i. (**C**,**D**) Putative miRNAs regulating the RISC-bound genes at 7 h p.i. and at 10 h p.i. Only miRNAs known to be expressed in alveolar macrophages^38^ and targeting at least five target genes are shown (*x* axis). Dots (*y* axis) represent the FC values of depleted and enriched RISC-bound genes targeted by each miRNA.

Putative miRNA target genes were over-represented among RISC-bound genes. About 80% of the statistically significant RISC-bound genes (Table 1) were putative target of at least one miRNA; they were distributed in similar proportions among enriched and depleted RISC-bound genes that were either upregulated or downregulated following infection in the whole cell (Fig. 5A,B). At 7 h p.i. only 185 (out of 968) predicted targets were enriched in RISC and concurrently downregulated in whole cell (upper left quadrant of the plot) while 411 targets were depleted in RISC but over-expressed in the whole cell (lower right quadrant of the scatter plot; Fig. 5A). This pattern is similar to what previously observed in cells infected by Lytic Adenovirus type 5 and suggests a likely trend towards “detargeting” of cellular mRNAs taking place during PRRSV infection^34^. This effect was no longer visible at 10 h p.i. when most target genes were found in RISC-depleted genes with FC < 1 in the whole cell (lower left plot) and only one target was found among RISC-enriched genes (upper left plot; Fig. 5B). As expected, most RISC-bound genes were potentially modulated by multiple miRNAs (multiplicity of miRNAs) at both times (Fig. 5C,D). As well, each miRNA recognized several target genes (cooperativity of miRNAs) as visible from Supplementary Tables S7 and S8).

#### Evidence for miRNA-mediated regulation of the IFN response

**Figure 6:**
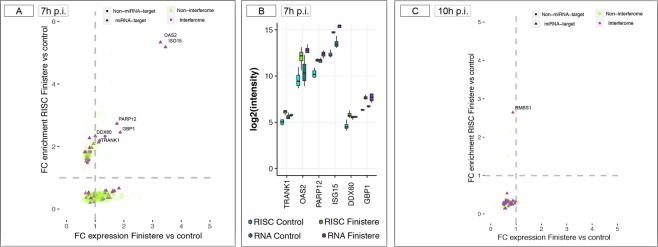
RISC-bound Interferon Regulated Genes (IRGs). (**A**) Distribution of IRGs within the 1,241 RISC-bound genes at 7 h p.i. The scatter plot depicts changes in mRNA levels in the whole cell (*x* axis) and in the RISC compartment (*y* axis). IRGs (violet dots) were identified using the interferome v2.0 (http://interferome.its.monash.edu.au/interferome/) database. Shape of dots indicates whether each IRG was targeted by at least one miRNA (triangle) or not-targeted (round). (**B**) RISC-enriched IRGs that were targeted by at least on miRNA and displayed positive FC values in the whole cell. (**C**) Distribution of IRGs within the 141 RISC-bound genes at 10 h p.i.

We looked for interferon-regulated genes (IRGs) among RISC-bound genes using the Interferome v2.01 database, which enables the identification of individual IRG or IRG signatures from high-throughput data sets^41^. In total 61 IRGs were identified using the following database criteria: specie (*Homo sapiens*), type of IFNs: all, organ system: lung, category of cell: alveolar macrophages. At 7 h p.i., these IRGs represented both enriched and depleted RISC-bound genes with either positive or negative FC value in the whole cell (Fig. 6A). Six IRGs were enriched in RISC and displayed positive FC values in the whole cell; among them, *OAS2* (targeted by miR-132-3p, miR-335-5p and miR-7-5p), *ISG15* (targeted by miR-146a-5p and miR-1-3p), and *GBP1* (targeted by miR-124-3p) were upregulated in the whole cell with statistical significance (Fig. 6B). At 10 h p.i., these three genes remained upregulated DEGs in the whole cell but, as the majority of other IRGs, were no longer statistically significantly bound to RISC (Fig. 6C). The qPCRs performed in an independent panel of PAMs confirmed this trend, with the exception of *OAS2* that showed a relative increase of RISC-bound amounts vs. the whole cell at 10 h p.i. (Table 2). Moreover, qPCRs put in evidence a stronger upregulation of *IFNB1* at both 7 h and 10 h p.i. compared with the values found by microarrays in the main experiment, with a ratio of RISC-bound amounts vs. the whole cell around 0.2 (Table 2). The latter observation is in line with the RISC depletion of *IFNB1* found by anota analysis, which however did not reach statistical significance.

**Figure 7:**
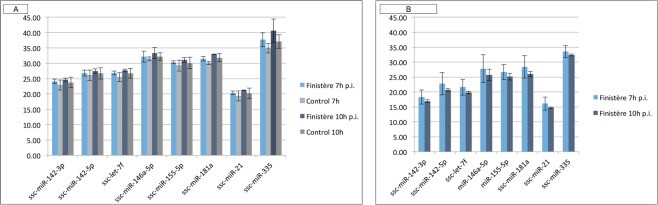
RT-qPCR profiling of a panel of eight miRNAs in PAMs. Average Ct values are shown for the whole cell (**A**) and the RISC compartment. (**B**) All RT-qPCR reactions were prepared from equal input RNA amounts.

Using residual RNAs from PAMs of the main experiment, we profiled by RT-qPCRs a panel of eight porcine miRNAs including some of the best-known innate immunity modulators (Fig. 7). In the whole cell, we found elevated inter-individual variability and no evidence of significant differential expression between uninfected and infected samples at both time points, although a trend towards down-regulation could be observed at both 7 h and 10 h p.i. (Fig. 7A). The relative levels of expression of the eight miRNAs reflect well their expected abundance as previously observed in uninfected bovine alveolar macrophages^38^ as well as upon infection with *Mycobacterium bovis*^42^.

As expected, in the RISC compartment all the eight miRNAs were better represented than in the whole cell (between 3 and 8 Ct of difference), supporting their functionality in regulating the RISC-bound transcriptome. In line with the results in the whole cell, their average abundance levels in the RISC of infected cells were similar between 7 h and 10 h p.i., with a decrease of inter-individual variability between 7 h and 10 h p.i. and only a trend towards increase in abundance at 10 h p.i. (Fig. 7B). Of note, ssc-miR-335 was barely detectable in the whole cell but highly enriched in the RISC compartment (Fig. 7B).

#### miR-335-5p has no detectable effects on surface markers, cytokine expression and PRRSV replication

**Figure 8:**
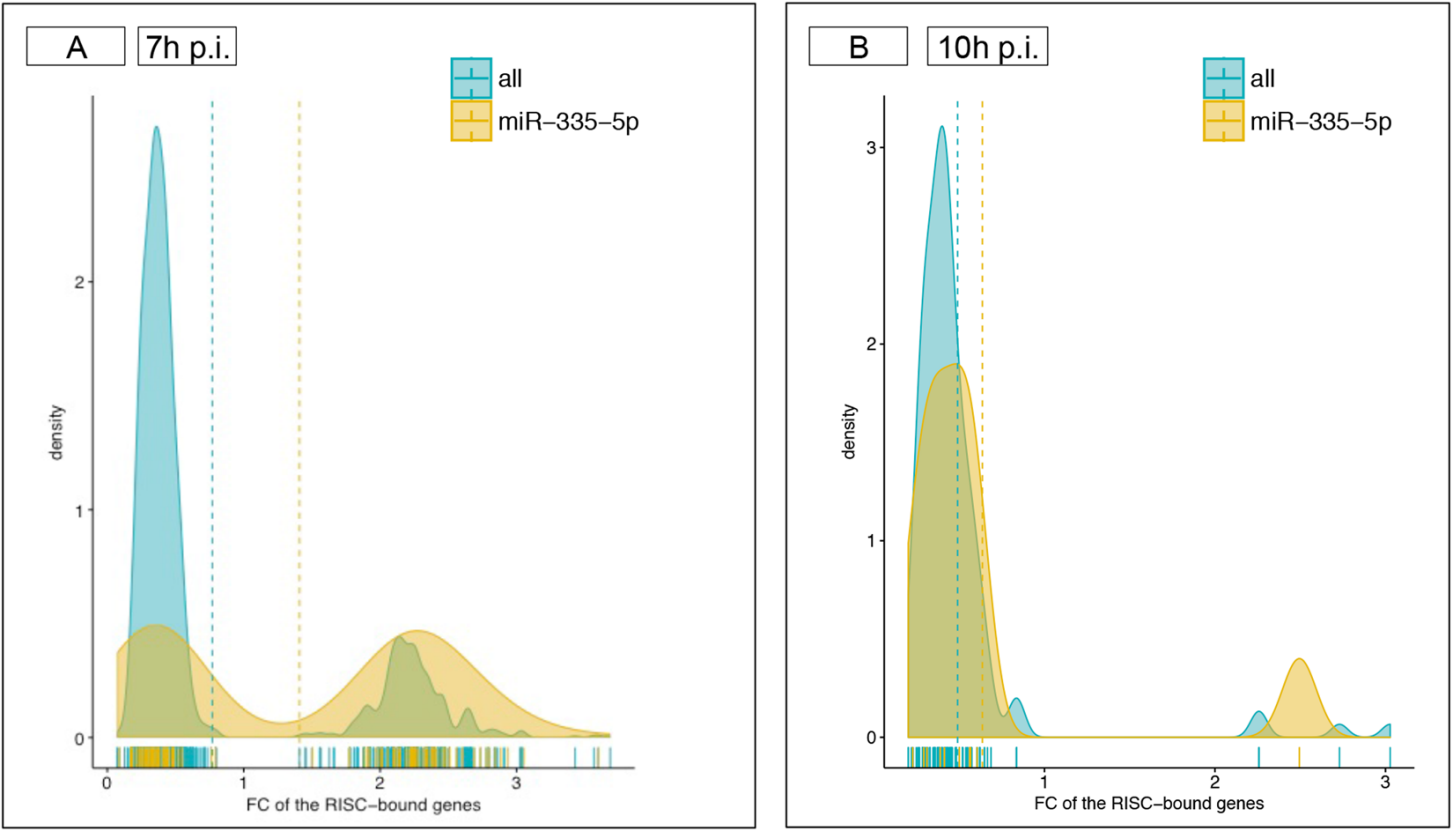
Putative targets of miRNA-335-5p. (**A**) The density plot visualizes the FC values of RISC-bound genes targeted by miR-335-5p (orange) compared to the FC values of the RISC-bound genes targeted by other miRNAs (blue) at 7 h p.i. Due to the similar proportion of enriched (n = 62) and depleted (n = 54) target genes of miR-335-5p at 7 h p.i., a bimodal distribution was observed. (**B**) The density plot visualizes the FC values of the 10 RISC-bound genes targeted by miR-335-5p (orange) compared to the FC values of the RISC-bound genes targeted by other miRNAs (blue) at 10 h p.i.

The miR-335-5p ranked second in term of total number of predicted gene targets in PAMs (116 in total) but was by far the miRNA with most targets (62) among the genes found enriched in the RISC at 7 h p.i. (Fig. 5C). For comparison, miR-26b-5p was predicted to target only 27 enriched RISC-bound genes out of its 134 total targets, and miR-26a-5p was predicted to target 5 out of 27 total targets enriched RISC-bound genes (Supplementary Table S7). The similar proportion of enriched (n = 62) and depleted (n = 54) target genes of miR-335-5p in RISC at 7 h p.i. determined a bimodal distribution, while for other miRNAs the excess of depleted targets determined a narrow and high peaked curve with its maximum <1 (Fig. 8A). The bimodal pattern of miR-335-5p targets is lost at 10 h p.i. (Fig. 8B). Finally, the 62 enriched targets of miR-335-5p at 7 h p.i. included genes characterized by high cooperativity of miRNA binding (e.g. *IGF1R*, insulin like growth factor 1 receptor, targeted by 30 miRNAs) as well as 18 genes targeted by miR-335-5p alone (Supplementary Table S7). The 62 genes represented several categories of proteins acting in transcriptional, translational and cell fate regulation across several metabolic pathways (Supplementary Fig. S5). Among these genes, *OAS2* is an IRG, and *TRANK1* (tetratricopeptide repeat and ankyrin repeat containing 1) has been identified as a non-classical IFN-stimulated gene^43^. Furthermore, *FOXO4* and *GATA*5 were two of the three top regulators TFs inferred for the whole set of 325 enriched RISC-bound genes (Supplementary Fig. S4).

We considered the above evidence as strongly suggestive of miR-335-5p functionality in the regulation of early PRSSV response. Given the high number of putative target genes of miR-335-5p, we decided to carry out miR-335-5p mimics experiments in the primary PAM, specifically to test how miR-335-5p modulates the expression of a panel of surface markers and cytokines, and the cell response to PRRSV infection (Supplementary Figs S6 and S7).

We first tested a broad range of miRNA mimics concentrations (10, 25, 35, 50 nM) and different times post-transfection in primary PAMs. The condition of 50 nM in miRNA mimics concentration with 30 h of transfection time yielded 70–80% of transfected cells with a dead rate of only 15–20% (Supplementary Fig. S6A). We used these conditions to measure the surface expression of CD80/86, MHCII and CD163 markers and the expression of a panel of cytokines (*IFN-α*, *IFN-γ*, *TNF-α*, *IL-10*, *1L-12*, *IL-1B*, *IL-6* and *IL-8*). The levels of MHCII and CD80/86 increased as an aspecific result of transfection (Supplementary Fig. S6B,C) and only slight changes, indistinguishable from the miRNA control, were found for individual cytokines (e.g. Supplementary Fig. S6D). A global analysis of cytokine expression by PCA demonstrated a change in inflammatory cytokines under miRNA mimics conditions occurring in PAMs with evidence of correlation with surface markers (not shown), suggesting that, the manipulation of primary PAMs by miRNA mimics induces a state similar to M1 polarization^44^. Furthermore, PRRSV replication was inhibited in transfected PAMs and a decreased expression of the main PRRSV receptor CD163 was observed (Supplementary Fig. S7). These data imply that, with this PRRSV strain and at early times post- infection, the immune activation triggered by a miRNA mimics manipulations hampers the possibility to detect faint modulatory effects of miRNA regulation in primary PAMs.

## Discussion

Our results show that the miRNA-targeted transcriptome of PAMs is modulated early following infection with the PRRSV Finistère strain, and that this effect decreases fast. At 7 h p.i., the number of enriched RISC-bound genes largely exceeded the number of DEGs in the whole cell, and decreased dramatically just three hours later, when cells were fully viable and sustaining PRRSV proliferation (Fig. 1 and Table 1). This result is also supported by the analysis of a parallel experiment (data not shown) with Lena, a highly virulent PRRSV-1.3 strain^8^. At 7 h p.i., Lena’s titres were higher than those of Finistère at 10 h p.i. (Supplementary Fig. S8) and at both times we could not identify any RISC-enrichment of host genes. We cannot formally exclude that specific interactions could occur at later post-infection times in PRRSV-infected PAMs. However, in designing this experiment we considered that long-term cultures of infected cells inevitably maximize levels of cytopathic effect. As observed in other host-virus systems, this would undoubtedly have the potential to influence cellular gene expression^45^.

The alterations of the miRNA-targeted transcriptome induced by PRRSV at 7 h p.i. involved genes representing a vast array of protein types and biological functions (Fig. 6). Such alterations had a mild impact on the transcriptome, as most RISC-bound genes were not significantly altered (DEG) in the whole cell. Moreover, we found no evidence for any of the validated miRNA-mRNA interactions reported for PRRSV^26^. The miRNAs identified by our approach included all of those reported to modulate host immune responses to different PRRSV strains, but with different predicted RISC-bound targets. For example, miR-30c has been described as a negative regulator of IFN-I signalling by targeting *JAK1* (Janus kinase 1) and *IFNAR2* (interferon alpha and beta receptor subunit 2), resulting in the enhancement of PRRSV infection by type 2 PRRSV strains^46,47^, but neither *JAK1* nor IFNAR2 were found RISC-bound or DEG in our study with the low virulent Finistère strain. Similarly, we did not find evidence of targeting of the *CD163*, the main PRRSV receptor (which is not DEG at both 7 h and 10 h p.i.) by miR-181^48^. This however is not surprising, given that most of these studies were carried out with different PRRSV genotypes and later times post-infection.

An emerging pattern was a likely buffering effect of miRNAs on interferon-regulated genes^49^. We found no evidence for targeting of the *IFNB1* gene itself, despite the fact that interferon beta can be modulated by miRNAs which are abundant in the macrophage (miR-26a, miR-34a, miR-145 and let-7b)^50^. However, six of the IRGs upregulated in the whole cell at both 7 h and 10 h p.i. were enriched in the RISC at 7 h p.i. (Fig. 6 and Table 2). These findings are in line with a pervasive action of miRNAs in the regulation of the IFN response emerging in literature^49^ and suggest a buffering effect of the miRNAs on IFN Type I signaling in PAMs occurring only at 7 h p.i. In addition, the RISC-bound genes enriched at 7 h p.i. included several examples of ‘expected’ targets of miRNAs that are known key modulators of innate immunity responses^49,51–53^. For example, miR-155-5p (having 16 putative targets at 7 h p.i.) can indirectly modulate type I IFN signal transduction by targeting *SOCS1* (suppressor of cytokine signaling 1), a negative regulator of the JAK-STAT pathway^54^; this regulation has been described in several cells including macrophages^55^. *SOCS1* was effectively enriched in RISC (FC 2.4) and was a putative target of other six miRNAs expressed in alveolar macrophages (miR-19a-3p, miR-19b-3p, miR-30b-5p, miR-30c-5p, -let-7i-5p, miR-122-5p).

Furthermore, qPCR results (Fig. 8) strongly suggest that, at least early during PRRSV infection, the miRNA-mediated regulation of host response largely relies on miRNAs that are expressed and functional in normal cell physiological conditions. This pattern is similar to what previously observed for HIV-1 virus at early times of infection^45^ and is indirectly confirmed by several host-pathogen interaction studies in which the fold changes detected for miRNAs are usually small and hardly reproducible across different experiments^26,45,56^. It should also be noted that we did not find evidence of downregulation of genes involved in the miRNA biogenesis pathway (Ago, Drosha and Dicer), despite it has been reported that the non-structural proteins of PRRSV can target Ago2 to favour viral infection^57^.

Finally, despite the abundance of putative targets of miR-335-5p in RISC-bound genes, we could not validate a role for this miRNA in the response of alveolar macrophages to PRRSV. The absence of detectable phenotypes effects on macrophage function and levels of PRRSV infection cannot be solely accounted to the reduced sensitivity of miRNA mimics in PAMs. The miR-335-5p has been reported as one of the miRNAs down-regulated in PBMCs from HIV-1 and HIV-2 infected patients^58^ and for having a role in the pathogenesis of influenza in the respiratory tract of dogs^59^. However, a recent atlas has shown that this miRNA locus is expressed at low levels in most tissues and cell types of the lung tract^60^. As coexpression is one of the fundamental criteria for functionality of predicted miRNA-mRNA interactions^40^, miR-335-5p may have negligible effects despite its relative strong enrichment in RISC, especially given that most of its putative target sites are recognized by several miRNAs highly expressed in these cells.

Overall, our results do not point to specific miRNA-driven mechanisms regulating the early response to infection with this PRRSV 1.1 strain. Instead, they indicate that the miRNome expressed by steady-state PAMs reacts promptly to counterbalance the stress represented by a viral infection by modulating the early immune response signaling cascade that leads to transcription factor activation and changes in the cell transcriptome (Fig. 3). As infection proceeds *in vitro*, the pervasive modulation operated by miRNAs is presumably overpowered by the innate immune responses, and other evasive mechanisms adopted by PRRSV^61^ would prevail on miRNAs. We also argue that the several miRNA-mRNA interactions described for different PRRSV strains^26^ may have a likely impact in the case of natural PRRSV infections, when different PAM subpopulations of the respiratory tract progressively encounter the virus.

## Methods

### Isolation, culture and infection of porcine alveolar macrophages

Four 7-week-old specific-pathogen-free (SPF) Large White piglets, obtained from the experimental pig herd of Anses^29^, were euthanized to collect the lungs. Bronchoalveolar lavages (BAL) were performed by infusing around 1L of warm, sterile PBS instilled in the lungs by the trachea. The recovered BAL fluid was centrifuged to purify the porcine alveolar macrophages (PAMs). From these four animals, four batches of around 10^9^ cells were collected from each animal and cryopreserved in liquid nitrogen in fetal bovine serum (FBS) containing 10% DMSO until cell culture. The low virulent PRRSV Finistère strain (also named as FR-2005-29-24-1) was propagated and titrated on PAMs for four passages and concentrated by centrifugation for 30 min at 5000 × g using Amicon® Ultra-15 centrifugal filter units 30 K (Merck Millipore, Billerica, MA, USA).

Frozen PAMs were rapidly thawed and immediately washed in PBS. For each experimental condition, 10^8^ PAMs were pre-incubated at 37 °C with 5% CO_2_ for 24 h in 175 cm^2^ flask in RPMI 1640 medium supplemented with 15% of gamma irradiated FBS, 5% of serum collected from SPF pig, 2 mM of L-Glutamine and 200 U/ml of penicillin*-*streptomycin. The four batches of cells (biological replicates) were then PRRSV-infected with the Finistère strain using a multiplicity of infection (MOI) of 2 for either 7 h or 10 h, in two technical replicates per time. Control (mock-infected) PAMs were simultaneously cultured for each condition (one technical replicate per time). At either 7 h or 10 h p.i. the culture medium was removed, PAMs were washed with PBS, harvested by scraping on ice in cold PBS supplemented with 5 mM EDTA and collected by centrifugation (5 min at 200 × g, at 4 °C). Cells were lysed using the lysis buffer of the Pierce IP classic kit (Thermo Scientific) supplemented with protease inhibitor (Roche) and RNase inhibitor (Thermo scientific). All cell lysates were stored at −80 °C.

The same experimental scheme was implemented on remaining PAMs using the high virulent PRRSV-1.3 Lena strain (Supplementary Fig. S8). The virus was propagated and titrated on PAMs for seven passages, and did not require concentration by centrifugation for the required MOI (2). Moreover, because all RISC-bound RNA was used for RISC-IP and microarray hybridizations (see below), additional 4 animals from the same SPF herd were sacrificed for obtaining an average of 500 millions PAMs per animal. Of these, 250 million cells were infected *in vitro* with the Finistère strain (MOI = 2) and the remaining ones used as controls.This additional material was used for the qPCR of a panel of 8 genes (Table 2) and for miRNA mimics experiments (Supplementary Figs S6 and S7).

For immunofluorescence staining of infected cells, PRRSV infection of PAMs from two of the four batches was reproduced with the same cell culture and infection conditions to evaluate average levels of infection with three technical replicates (Fig. 1 and Supplementary Fig. S8). At 7 h or 10 h p.i., the culture medium was removed and conserved at −80 °C for virus titration. Cells were gently washed twice with PBS-0.1% tween 20 before fixation in cold acetone 80% for 30 min. To visualize infected cells, PAMs were stained for 45 min, first with monoclonal mouse anti-PRRSV (N protein) antibody at the dilution of 1:100 (Bio-X Diagnostics, Jemelle, Belgium), then by anti-mouse IgG Alexa 488-labelled at the dilution of 1:500 (Life technologies, Carlsbad, CA, USA). Additional nuclei staining for 5 min with Hoechst solution at the dilution of 1:10 (Sigma-Aldrich, St Louis, USA) was applied in order to visualize the all cells. Microscopy observations were performed with an Olympus IX-71 inversed fluorescence microscope (Scop Pro, Itteville, France). Images were acquired by an EXI Aqua camera (QImaging, Surrey, Canada) using Image Pro-Plus software (Media Cybernetics). PRRSV was titrated on freshly cultured PAMs in 96-well plates using serial dilutions of the culture medium collected from infected cells. The viral titre was calculated using the Karber’s method after observation of cytopathic effects in each well. The results were expressed as the mean of two biological replicates and two technical replicates for each condition.

The experimental protocols on animals were approved by the “Animal health and welfare” committee of ANSES. The method of collection of porcine alveolar macrophages was carried out in accordance with European and French legislation, ethical and welfare recommendations at ANSES Ploufragan facilities (Agreement number C-22-745-1).

### Immunoprecipitation of the RISC complex (RISC-IP)

RISC-IP was carried out using monoclonal anti-pan-Argonaute antibody 2A8 (Merck Millipore) that recognizes Ago1, Ago2, Ago3 and Ago4^62^ and has been previously used in a study on sheep^63^. We used the Pierce IP classic kit (Thermo Scientific) according to manufacturer’s specifications with some modifications detailed on the project website (http://ago.rockefeller.edu^64^. Protein A/G agarose beads were pre-incubated five hours at 4 °C with polyclonal rabbit anti-mouse IgG (Jackson Immunoresearch) to increase their avidity and over-night at 4 °C with Anti-pan Ago clone 2A8. The thawed cell lysates were incubated with the antibodies-protein A\G Plus agarose resin complex followed by washing five times with IP lysis/wash buffer from the IP classic kit. An aliquot of the thawed cell lysate was set aside for extraction of total (whole cell) RNAs.

For Western Blot analysis, proteins of whole cell lysate and flow through fractions from infected and non-infected PAMs were separated by SDS-PAGE and transferred onto nitrocellulose membranes. The membranes were incubated in blocking solution (1X PBS, 0.05% Tween 20 supplemented with 5% milk) for 1 h. Blots were incubated over-night at 4 °C with the Anti-pan Ago clone 2A8 (Merck-Millipore). The membranes were rinsed with PBS containing 0.05% Tween 20 and incubated for 1 h with a goat anti-mouse HRP-conjugated secondary antibodies diluted in blocking solution. The membranes were rinsed, and immunodetection was performed by using an enhanced chemiluminescence (ECL) substrate (BioRad, France). An Anti-alpha Tubulin (acetyl K40) antibody [6-11B-1] served as a control (Supplementary Fig. S1).

### RNA extractions

Total RNA and RISC-bound RNAs were extracted and purified using TRIzol LS Reagent (Life Technologies) and further purified using the RNeasy MinElute clean up kit (Qiagen) according to the manufacturers’ recommendations. RNA yield and purity were monitored by spectrophotometry (NanoDrop ND-1000). RNA integrity was assessed using an Agilent 2100 Bioanalyzer and the RNA 6000 pico kits. The average RIN (RNA Integrity Number) values of extracted RNAs used for RIP-Chip (main experiment) were 9.3 (non-infected samples) and 7.5 (infected samples). For the second batch of PAMs, the average RIN values were 7.9 (non-infected samples) and 7.1 (infected samples).

### Microarray hybridizations

Transcriptome profiling was performed using a pig custom 8 × 60 K microarray (Agilent-037880/INRA Suscrofa60K v1 platform; GEO platform Number: GPL16524). Cyanine-3 (Cy3) labeled cRNAs were prepared from whole cell and RISC-IP RNAs using the One-Color Low Input Quick Amp Labeling kit (Agilent Technologies, Santa Clara, CA, USA), The starting RNA amount was 50 ng and 10 ng for whole cell and RISC-IP RNAs, respectively. For both types of RNA samples specific activities and cRNA yields were measured using the NanoDrop ND-1000 (Thermo Fisher Scientific, Waltham, MA, USA). Subsequently, for each sample 600 ng of Cy3-labeled cRNA (specific activity >9.0 pmol Cy3/µg of cRNA) were fragmented at 60 °C for 30 minutes in a volume of 25 µl containing 25x of Agilent Fragmentation Buffer and 10x of Agilent Blocking Agent. Afterwards, 25 µl of 2x Agilent Hybridization Buffer were added to the fragmentation mixture and hybridized to the array following the recommended protocol. After hybridization, microarrays were washed for 1 minute at room temperature with the GE Wash Buffer 1 (Agilent Technologies) and 1 minute at 37 °C using the GE Wash Buffer 2 (Agilent Technologies). The slides were then scanned using a G2565CA Scanner System (Agilent Technologies), using a scan protocol with a resolution of 3 µm and a dynamic range of 20 bit. The resulting.tiff images were analyzed with the Feature Extraction Software v10.7.3.1 (Agilent Technologies), using the GE1_107_Sep09 protocol.

### Probe contents and refinement of probe annotation

We used a custom porcine Agilent microarray enriched for immunity related genes as previously described^65^. In order to improve the annotation of the array, the 60-mer sequence probes were mapped onto the pig genome reference SusScorfa10.2 with TopHat aligner (v2.0.14). Only probes mapping to a unique position were further annotated by combining the porcine genome annotation provided by Ensembl and NCBI databases. The final number of unique porcine genes annotated in the array was 13,106.

### miRNA and mRNA quantification by RT-qPCR

Expression analysis of a panel of eight miRNAs (ssc-miR-21-5p, ssc-miR-335, ssc-miR-155-5p, ssc-miR-181a, ssc-miR-146a-5p, ssc-let-7f-5p, ssc-miR-142-5p and ssc-miR-142-3p) was carried using the miRCURY LNA™ microRNA PCR system (Exiqon). We could use the PCR primer sets from Exiqon, as all the miRNAs profiled in this study are fully conserved in human, cattle and pig with the exception of ssc-miR-155-5p, whose sequence differs from hsa-miR-155-5p and bta-miR-155 for a point mutation at position 12 (outside of the seed region). For reverse transcriptions, 10 ng of either total RNA or RISC-bound RNA were mixed with a synthetic RNA spike-in control (UniSp6) and cDNA was diluted 80 times in nuclease free water. The qPCR reactions (10 μl) were performed using ExiLENT SYBR Green master mix (Exiqon) using a QuantStudio 12 K Flex Real-Time PCR System (Applied Biosystems). Amplification plots were analyzed using the QuantStudio 12 K Flex software, both for determination of Ct values and for melting curve analysis. For the qPCRs of a panel of 8 coding genes (*FOXO4*, *IFITM1*, *GBP1*, *ISG15*, *HMGB1*, *OAS2*, *STAT1* and *IFNB1*) we used the RNAs extracted at 7 h and 10 h p.i. from the PAMs of the second *in vitro* experiment (4 animals). We used standard protocols for primer design, cDNA synthesis and qPCR, as previously described^56^. Primer sequences were designed using the PrimerExpress 2.0 software (Applied Biosystems, Thermo Fisher) and were: *FOXO4*, F: TGGAAAACCTGGAGTGTGACAT, R: TCCAGTCCTTCGCCTCCAT; *IFITM1*, F: CGCCAAGTGCCTGAACATCT, R: GTGGCTCCGATGGTCAGAA), *GBP1*, F: CCTGGAGAACTCACTCAAGCTTAAG, R: GAGTCGAGGCAGGTTAAAATTTTT; *ISG15*, F: GAGGACCTGTCGCCAAAGC, R: CCCAGCATCTTCACCTTCAGTT; *HMGB1*, F: CGAAAAGGATATTGCTGCATACC, R: TGACTCCCTTTTTTGCTGCAT; *OAS2*, F: TCCAAGATGGGAGTGGATATCC, R: CACAGCTTCCTGGTGTCTGTACTG; *STAT1*, F: CCTGTTGCGGTTCAGTGAGA, R: GGTTCAACTGCATGGAAGTAAGG; and *IFNB1*, F: TCTCTAGCACTGGCTGGAATGA, R: CTGCCCATCAAGTTCCACAA.

### miRNA mimics

The miRNA mimics was carried out using the miRCURY LNA™ microRNA (Exiqon, Qiagen) reagents, and included: hsa-miR335-5p (5′-UCAAGAGCAAUAACGAAAAAUGU-3′), hsa-miR335-5p-FAM (5′ FAM fluorescently labeled for assessment of transfection efficiency) and cel-miR-39-3p (5′-UCACCGGGUGUAAAUCAGCUUG-3′) as negative control. PAMs were seeded in 48 well plates (100,000 cells per well) in the RPMI 1640 complete medium (see above), and pre-incubated overnight at 5% CO_2_, 37 °C. Prior transfection, cells where washed twice with RPMI alone and placed in transfection medium (RPMI 1640 medium supplemented with 10% inactivated FBS and 2 mM L-glutamine). PAMs were transfected for 30 h at 5% CO_2_, 37 °C in a transfection medium with DharmaFECT4 (Dharmacon, USA) diluted in 1X siRNA buffer and containing each miRNA mimics, whose concentrations were first tested (between 35–50 nM for 10^6^ cells/ml). Negative control cells (referred as Mock) were incubated with siRNA buffer and transfection medium alone, whereas transfection control cells (referred as D4) were incubated with siRNA buffer, DharmaFECT4 and transfection medium. For PRRSV infections (Finistère strain) transfected PAM were washed twice with RPMI alone and infected at MOI = 0.01 in complete medium for 35 h at 5% CO_2_, 37 °C.

Transfected PAMs were harvested using 1x Trypsin to perform intracellular staining using flow cytometry. Cell viability was evaluated with DAPI staining (Sigma-Aldrich) and the percentage of miRNA transfected PAM was expressed by hsa-miR335-5p-FAM+ labelled cells percentage. The list of antibodies for the staining of surface markers is provided in Supplementary Table S9A. For CD80/CD86 staining, the protein expressed by the human recombinant CD152 gene genetically fused with the Fc portion of the murine IgG2a sequence was used (Ancell, Bayport, MN, USA). Cells were stained in blocking solution (PBS/EDTA supplemented with 5% Horse Serum and 5% Swine Serum). Antibodies were added to the blocking solution for 30 min on ice and then washed in PBS/EDTA with 2% FBS. Staining were made in two steps, the uncoupled primary antibodies of different species/isotypes followed by the secondary species/isotype specific fluorochrome-coupled antibodies and by DAPI staining for dead/live cells. Signals were acquired using an LSRFortessa cytometer and the Diva software (Becton Dickinson, Franklin Lakes, New Jersey). The FlowJo software (version X.1.0, Tree Star, Ashland, OR, US) was used to analyze markers expression. For intracellular virus detection, an intracellular staining was performed using BD Cytofix/Cytoperm™ (Becton-Dickinson, Belgium), according to the manufacturer’s instructions using an antibody against viral N protein (clone BIO 276, Bio-X Diagnostic).

The supernatants were collected and used for the Cytokine Bead Assay (CBA) as previously described using the capture and detection antibodies combinations described in Supplementary Table S9B.

### Statistical analysis of microarray data

All the transcriptome pre-processing, normalization and statistical analysis steps were carried out using several Bioconductor packages in R programming language (version 3.02). Firstly, the data’s quality was checked with the array Quality Metrics package^66–67^. Subsequently, probes showing any of the following Agilent flags (gIsFeatPopnOL = 1, gIsBGNonUnifOL = 1, gIsFeatNonUnifOL = 1) were removed from the analysis. The raw intensity values were then log2 transformed and a between-array normalization was performed using the 75th percentile. The normalization procedure was done separately for the whole cell arrays and the RISC-bound arrays. PCA was performed with FactoMineR and ade4 packages (version 1.23), in order to establish whether a particular array contributed markedly to variability in the gene expression data (i.e. whether it retained most of the information).

Differentially expressed genes in the whole cell were identified by using the limma package^68^; version 3.14.4. At each time point, the statistical linear model included the treatment (PRRSV/Control) and animal as a fixed effect. To make the analysis more robust and control more strictly for the false discovery rate (FDR), the *p*-values were corrected for Benjamini-Hochberg method with a threshold of adjusted p < 0.05, as a compromise between the unadjusted analysis and the Bonferroni procedures. The RIP-Chip enrichment analyses were performed with the anota Bioconducor R package^32^. This package implements analysis of partial variance (APV) combined with a variance shrinkage method for estimating random error. Genes with an APV adjusted p value of <0.05 were considered enriched or depleted in the RISC. Genes with unrealistic slopes (p slope <001, and >2 or <−1) were filtered out.

### Generation of a functional annotation map from a list of genes

We first selected genes with at least one of their probe sets being significantly expressed (q < 0.05) between treatments using anota method for the RISC-bound genes or limma for the whole cell. Multiple probes for the given genes were then summarized based on their median expression value, using the avereps function in limma R package.

The GO term and the Kyoto Encyclopaedia of Genes and Genomes (KEGG) enrichment analyses of genes were performed using Cytoscape V2.7 (http://cytoscape.org/) with the ClueGo V1.3 plug-in^69^. ClueGO determines the distribution of the gene list for the various GO terms and pathways. The *p*-value was calculated using right-sided hypergeometric tests and the Benjamini-Hochberg correction for multiple testing (FDR < 0.05). Together with this FDR threshold, a high kappa value (0.4) enabled us to precisely select GO terms enriched in highly connected genes.

### Use of iRegulon to identify TFs involved in biological processes

The iRegulon computational method was used to identify TFs within the set of genes and target genes^70^. iRegulon searches the regulatory sequences around each gene in order to detect enriched TF motifs using a database of nearly 10,000 TF motifs. iRegulon links enriched motifs to candidate TFs and determines the optimal subset of direct target genes.

### miRNAs and their putative gene targets

For each miRNA in miRBase 21, we assembled a comprehensive list of all experimentally validated target genes (using the multiMiR v2.1 package in R (updated 12/22/2016)^71^. multiMiR is a comprehensive collection of predicted and validated miRNA–target interactions and their associations with diseases and drugs. It contains human and murine data from 14 external databases categorized into three components, including three validated miRNA–target databases (miRecords v4, miRTarBase v6.1 and TarBase 6), eight predicted miRNA–target databases (DIANA-microT-CDS v5, ElMMo v5, MicroCosm v5, miRanda, miRDB v5, PicTar v2, PITA v6 and TargetScan v6.2), and three disease- or drug-related miRNA databases (miR2Disease, Pharmaco-miR and PhenomiR v2). A target gene was considered to be experimentally validated when either luciferase assay, Western blot, CLASH, HITS-CLIP, or PAR-CLIP methods had been used to determine the specific miRNA-mRNA interaction^71^. Subsequently, we subtracted the set of miRNAs presenting at least one RISC-bound gene at 7 h p.i. or 10 h p.i. Lastly, we selected, among miRNAs with at least one RISC-bound gene, only those expressed in bovine alveolar macrophages^38^.

## Supplementary information

Supplementary Figures S1-S8

Tables S1, S2, S3, S4, S5, S6, S6, S7, S8, S9

## References

[1] Lunney JK, Benfield DA, Rowland RR (2010). Porcine reproductive and respiratory syndrome virus: an update on an emerging and re-emerging viral disease of swine. Virus Res.

[2] Zhou L, Yang H (2010). Porcine reproductive and respiratory syndrome in China. Virus Res.

[3] Snijder EJ, Kikkert M, Fang Y (2013). Arterivirus molecular biology and pathogenesis. J Gen Virol.

[4] Kuhn JH (2016). Reorganization and expansion of the nidoviral family Arteriviridae. Arch Virol.

[5] Allende R (1999). North American and European porcine reproductive and respiratory syndrome viruses differ in non-structural protein coding regions. J Gen Virol.

[6] Nelsen CJ, Murtaugh MP, Faaberg KS (1999). Porcine reproductive and respiratory syndrome virus comparison: divergent evolution on two continents. J Virol.

[7] Kappes MA, Faaberg KS (2015). PRRSV structure, replication and recombination: Origin of phenotype and genotype diversity. Virology.

[8] Stadejek T (2008). Definition of subtypes in the European genotype of porcine reproductive and respiratory syndrome virus: nucleocapsid characteristics and geographical distribution in Europe. Arch Virol.

[9] Lunney JK (2016). Porcine Reproductive and Respiratory Syndrome Virus (PRRSV): Pathogenesis and Interaction with the Immune System. Annu Rev Anim Biosci.

[10] Du T, Nan Y, Xiao S, Zhao Q, Zhou EM (2017). Antiviral Strategies against PRRSV Infection. Trends Microbiol.

[11] Burkard C (2017). Precision engineering for PRRSV resistance in pigs: Macrophages from genome edited pigs lacking CD163 SRCR5 domain are fully resistant to both PRRSV genotypes while maintaining biological function. Plos Pathog.

[12] Burkard, C. *et al*. Pigs Lacking the Scavenger Receptor Cysteine-Rich Domain 5 of CD163 Are Resistant to Porcine Reproductive and Respiratory Syndrome Virus 1 Infection. *J Virol* **92**, 10.1128/JVI.00415-18 (2018). 10.1128/JVI.00415-18 PMC6069206 29925651

[13] Bartel DP (2004). MicroRNAs: genomics, biogenesis, mechanism, and function. Cell.

[14] Pasquinelli AE (2012). MicroRNAs and their targets: recognition, regulation and an emerging reciprocal relationship. Nat Rev Genet.

[15] Bartel DP (2009). MicroRNAs: target recognition and regulatory functions. Cell.

[16] Krol J, Loedige I, Filipowicz W (2010). The widespread regulation of microRNA biogenesis, function and decay. Nat Rev Genet.

[17] Carthew RW, Sontheimer EJ (2009). Origins and Mechanisms of miRNAs and siRNAs. Cell.

[18] Rinck A (2013). The human transcriptome is enriched for miRNA-binding sites located in cooperativity-permitting distance. RNA Biol.

[19] Small EM, Olson EN (2011). Pervasive roles of microRNAs in cardiovascular biology. Nature.

[20] Kowarsch A, Marr C, Schmidl D, Ruepp A, Theis FJ (2010). Tissue-specific target analysis of disease-associated microRNAs in human signaling pathways. Plos One.

[21] Kowarsch A, Preusse M, Marr C, Theis FJ (2011). miTALOS: analyzing the tissue-specific regulation of signaling pathways by human and mouse microRNAs. RNA.

[22] Fischer S, Handrick R, Aschrafi A, Otte K (2015). Unveiling the principle of microRNA-mediated redundancy in cellular pathway regulation. RNA Biol.

[23] Cullen BR (2013). MicroRNAs as mediators of viral evasion of the immune system. Nat Immunol.

[24] Trobaugh DW, Klimstra WB (2017). MicroRNA Regulation of RNA Virus Replication and Pathogenesis. Trends Mol Med.

[25] Barnes D, Kunitomi M, Vignuzzi M, Saksela K, Andino R (2008). Harnessing endogenous miRNAs to control virus tissue tropism as a strategy for developing attenuated virus vaccines. Cell Host Microbe.

[26] Liu, F., Du, Y. & Feng, W. H. New perspective of host microRNAs in the control of PRRSV infection. *Vet Microbiol*, 10.1016/j.vetmic.2017.01.004 (2017). 10.1016/j.vetmic.2017.01.004 28161213

[27] Keene JD, Komisarow JM, Friedersdorf MB (2006). RIP-Chip: the isolation and identification of mRNAs, microRNAs and protein components of ribonucleoprotein complexes from cell extracts. Nat Protoc.

[28] Tan LP (2009). A high throughput experimental approach to identify miRNA targets in human cells. Nucleic Acids Res.

[29] Renson P (2017). Dynamic changes in bronchoalveolar macrophages and cytokines during infection of pigs with a highly or low pathogenic genotype 1 PRRSV strain. Vet Res.

[30] Genini S (2008). Genome-wide transcriptional response of primary alveolar macrophages following infection with porcine reproductive and respiratory syndrome virus. Journal of General Virology.

[31] Badaoui B (2014). RNA-sequence analysis of primary alveolar macrophages after *in vitro* infection with porcine reproductive and respiratory syndrome virus strains of differing virulence. Plos One.

[32] Larsson O, Sonenberg N, Nadon R (2011). anota: Analysis of differential translation in genome-wide studies. Bioinformatics.

[33] Dölken L (2010). Systematic analysis of viral and cellular microRNA targets in cells latently infected with human gamma-herpesviruses by RISC immunoprecipitation assay. Cell Host Microbe.

[34] Bellutti F, Kauer M, Kneidinger D, Lion T, Klein R (2015). Identification of RISC-associated adenoviral microRNAs, a subset of their direct targets, and global changes in the targetome upon lytic adenovirus 5 infection. J Virol.

[35] Yanai H (2009). HMGB proteins function as universal sentinels for nucleic-acid-mediated innate immune responses. Nature.

[36] Badaoui B (2013). Pig immune response to general stimulus and to porcine reproductive and respiratory syndrome virus infection: a meta-analysis approach. BMC Genomics.

[37] Peterson SM (2014). Common features of microRNA target prediction tools. Front Genet.

[38] Vegh P (2013). Profiling microRNA expression in bovine alveolar macrophages using RNA-seq. Vet Immunol Immunopathol.

[39] Londin E (2015). Analysis of 13 cell types reveals evidence for the expression of numerous novel primate- and tissue-specific microRNAs. Proc Natl Acad Sci USA.

[40] Berezikov E (2011). Evolution of microRNA diversity and regulation in animals. Nat Rev Genet.

[41] Rusinova I (2013). Interferome v2.0: an updated database of annotated interferon-regulated genes. Nucleic Acids Res.

[42] Vegh P (2015). MicroRNA profiling of the bovine alveolar macrophage response to Mycobacterium bovis infection suggests pathogen survival is enhanced by microRNA regulation of endocytosis and lysosome trafficking. Tuberculosis (Edinb).

[43] Ignatius Irudayam J (2015). Characterization of type I interferon pathway during hepatic differentiation of human pluripotent stem cells and hepatitis C virus infection. Stem Cell Res.

[44] Arora S, Dev K, Agarwal B, Das P, Syed MA (2018). Macrophages: Their role, activation and polarization in pulmonary diseases. Immunobiology.

[45] Whisnant AW (2013). In-depth analysis of the interaction of HIV-1 with cellular microRNA biogenesis and effector mechanisms. MBio.

[46] Zhang Q (2016). MicroRNA-30c Modulates Type I IFN Responses To Facilitate Porcine Reproductive and Respiratory Syndrome Virus Infection by Targeting JAK1. J Immunol.

[47] Liu, F. *et al*. MicroRNA-30c targets the interferon-alpha/beta receptor beta chain to promote type 2 PRRSV infection. *J Gen Virol*, 10.1099/jgv.0.001166 (2018). 10.1099/jgv.0.001166 30382935

[48] Gao L (2013). MicroRNA 181 suppresses porcine reproductive and respiratory syndrome virus (PRRSV) infection by targeting PRRSV receptor CD163. J Virol.

[49] Forster SC, Tate MD, Hertzog PJ (2015). MicroRNA as Type I Interferon-Regulated Transcripts and Modulators of the Innate Immune Response. Front Immunol.

[50] Witwer KW, Sisk JM, Gama L, Clements JE (2010). MicroRNA regulation of IFN-beta protein expression: rapid and sensitive modulation of the innate immune response. J Immunol.

[51] Lee HM, Nguyen DT, Lu LF (2014). Progress and challenge of microRNA research in immunity. Front Genet.

[52] Li Y, Shi X (2013). MicroRNAs in the regulation of TLR and RIG-I pathways. Cell Mol Immunol.

[53] Taganov KD, Boldin MP, Chang KJ, Baltimore D (2006). NF-kappaB-dependent induction of microRNA miR-146, an inhibitor targeted to signaling proteins of innate immune responses. Proc Natl Acad Sci USA.

[54] Piganis RA (2011). Suppressor of cytokine signaling (SOCS) 1 inhibits type I interferon (IFN) signaling via the interferon alpha receptor (IFNAR1)-associated tyrosine kinase Tyk2. J Biol Chem.

[55] Androulidaki A (2009). The kinase Akt1 controls macrophage response to lipopolysaccharide by regulating microRNAs. Immunity.

[56] Mahjoub N (2015). A 2.5-kilobase deletion containing a cluster of nine microRNAs in the latency-associated-transcript locus of the pseudorabies virus affects the host response of porcine trigeminal ganglia during established latency. J Virol.

[57] Chen J (2015). Porcine Reproductive and Respiratory Syndrome Virus (PRRSV) Inhibits RNA-Mediated Gene Silencing by Targeting Ago-2. Viruses.

[58] Devadas, K. *et al*. Identification of Host Micro RNAs That Differentiate HIV-1 and HIV-2 Infection Using Genome Expression Profiling Techniques. *Viruses* **8**, 10.3390/v8050121 (2016). 10.3390/v8050121 PMC4885076 27144577

[59] Fu C, Luo J, Ye S, Yuan Z, Li S (2018). Integrated Lung and Tracheal mRNA-Seq and miRNA-Seq Analysis of Dogs with an Avian-Like H5N1 Canine Influenza Virus Infection. Front Microbiol.

[60] de Rie D (2017). An integrated expression atlas of miRNAs and their promoters in human and mouse. Nat Biotechnol.

[61] Wang R, Zhang YJ (2014). Antagonizing interferon-mediated immune response by porcine reproductive and respiratory syndrome virus. Biomed Res Int.

[62] Nelson PT (2007). A novel monoclonal antibody against human Argonaute proteins reveals unexpected characteristics of miRNAs in human blood cells. RNA.

[63] Takeda H, Charlier C, Farnir F, Georges M (2010). Demonstrating polymorphic miRNA-mediated gene regulation *in vivo*: application to the g+ 6223G− > A mutation of Texel sheep. RNA.

[64] Chi SW, Zang JB, Mele A, Darnell RB (2009). Argonaute HITS-CLIP decodes microRNA-mRNA interaction maps. Nature.

[65] Voillet V (2014). Muscle transcriptomic investigation of late fetal development identifies candidate genes for piglet maturity. BMC Genomics.

[66] Maisonnasse P, Bordet E, Bouguyon E, Bertho N (2016). Broncho Alveolar Dendritic Cells and Macrophages Are Highly Similar to Their Interstitial Counterparts. Plos One.

[67] Kauffmann A, Gentleman R, Huber W (2009). arrayQualityMetrics–a bioconductor package for quality assessment of microarray data. Bioinformatics.

[68] Smyth, G. K. Linear models and empirical bayes methods for assessing differential expression in microarray experiments. *Stat Appl Genet Mol Biol* **3**, Article 3, 10.2202/1544-6115.1027 (2004). 10.2202/1544-6115.1027 16646809

[69] Bindea G (2009). ClueGO: a Cytoscape plug-in to decipher functionally grouped gene ontology and pathway annotation networks. Bioinformatics.

[70] Janky R (2014). iRegulon: from a gene list to a gene regulatory network using large motif and track collections. Plos Comput Biol.

[71] Ru Y (2014). The multiMiR R package and database: integration of microRNA-target interactions along with their disease and drug associations. Nucleic Acids Res.

