# Supplementary material for: The miRNA-targeted transcriptome of porcine alveolar macrophages upon infection with Porcine Reproductive and Respiratory Syndrome Virus
Source: Sci Rep. 2019 Feb 28;9:3160. doi: 10.1038/s41598-019-39220-3 (PMC6395673; doi:10.1038/s41598-019-39220-3)
Supplement: Supplementary file 1 — Supplementary Figures S1-S8 [file 41598_2019_39220_MOESM1_ESM.pdf]

Supplementary Figures S1 – S8

The miRNA-targeted transcriptome of porcine alveolar macrophages upon infection with Porcine Reproductive and Respiratory Syndrome Virus.

Sophie Dhorne-Pollet[1,+], Elisa Crisci[1,+,$], Nuria Mach[1,+], Patricia Renson[2], Florence Jaffrézic[1], Guillemette Marot[3], Tatiana Maroilley[1,#], Marco Moroldo[1], Jérôme Lecardonnel[1], Fany Blanc[1], Nicolas Bertho[4,&], Olivier Bourry[2], and Elisabetta Giuffra[1,*]

[1]GABI, INRA, AgroParisTech, Université Paris Saclay, Jouy –en-Josas, 78350, France
[2]ANSES, Unité Virologie Immunologie Porcines, Ploufragan, 22440, France
[3]EA 2694 Biostatistiques, Université de Lille, Inria Lille Nord Europe, MODAL, Villeneuve d'Ascq, 59650, France
[4]Virologie et Immunologie Moléculaire, Institut National de la Recherche Agronomique, Université Paris-Saclay, Jouy-en-Josas, France
[+]these authors contributed equally to this work
*Corresponding author, email: elisabetta.giuffra@inra.fr

([$]) Current address: Department of Population Health and Pathobiology, College of Veterinary Medicine, North Carolina State University, Raleigh, NC, United States
([#]) Current address: Departments of Medical Genetics and Biochemistry & Molecular Biology, Alberta Children's Hospital Research Institute (ACHRI), Cumming School of Medicine, University of Calgary, Canada
([&]) Current address: PIPAE, BIOEPAR, INRA, ONIRIS, Nantes Atlantic National College of Veterinary Medicine, Nantes, 44307, France

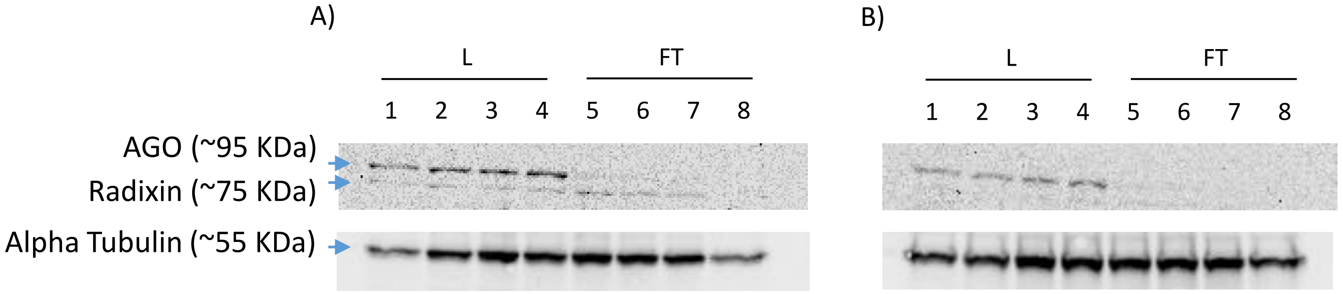

Figure S1. Efficiency of AGO2-RISC IP in alveolar macrophages. Western blot analysis showing comparable efficiency of Ago immunoprecipitation in whole cell lysate (L) and the relative impoverishment of Ago in the flow through (FT) fractions. Both fractions were obtained from PAMs of 2 animals (A and B) which were infected or not-infected with PRRSV. Lanes 1 and 5: control 7h; 2 and 6: infected 7h p.i., 3 and 7: control 10h; 4 and 8: infected 10h p.i. Arrows indicate Ago (~ 95 kDa), Radixin (~70 kDa) and alpha tubulin (~55 KDa). Note that Anti-pan Ago Antibody (clone 2A8) is known to cross-react with radixin (Nelson, P., et al. (2007). RNA. 13:1787–1792).

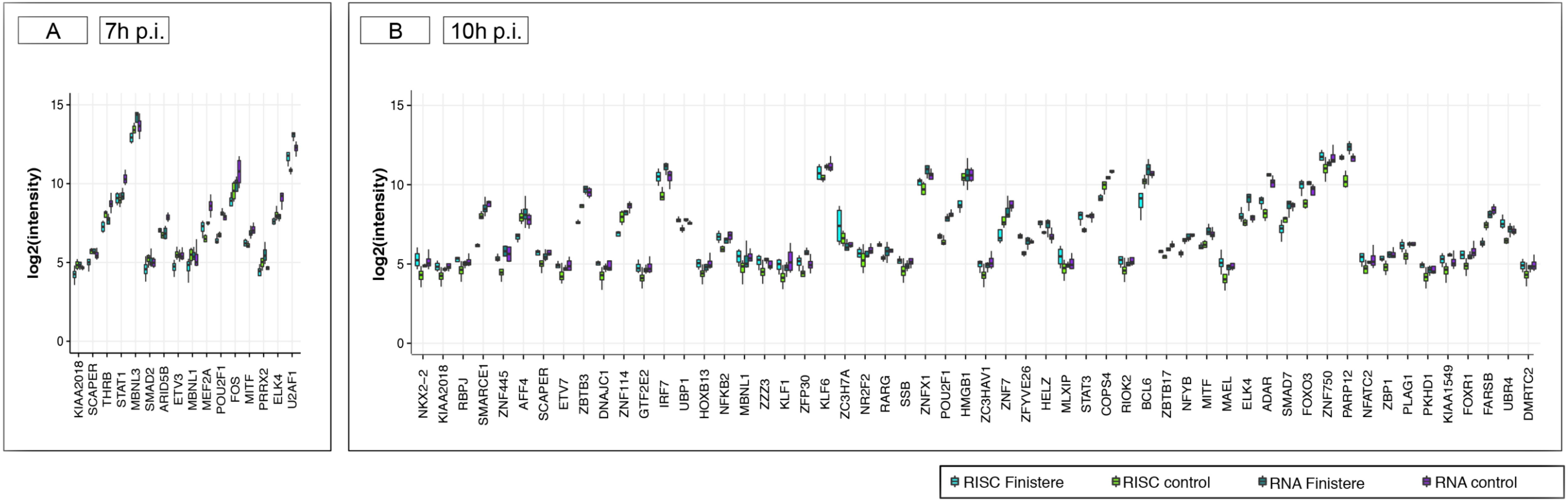

Figure S2. Transcription factors identified in the whole cell at 7h and 10h post-infection. Expression levels of the transcription factors (TFs) identified among DEGs in the whole cell at 7h p.i. (A) and at 10h p.i. (B) Both the expression in the RISC and in the whole cell is depicted. Boxes show median and interquartile range, and whiskers indicate 5th to 95th percentile. In each plot, RISC component after virus infection (cyan), RISC component control (light green), whole cell after virus infection (dark green) and control in the whole cell (violet).

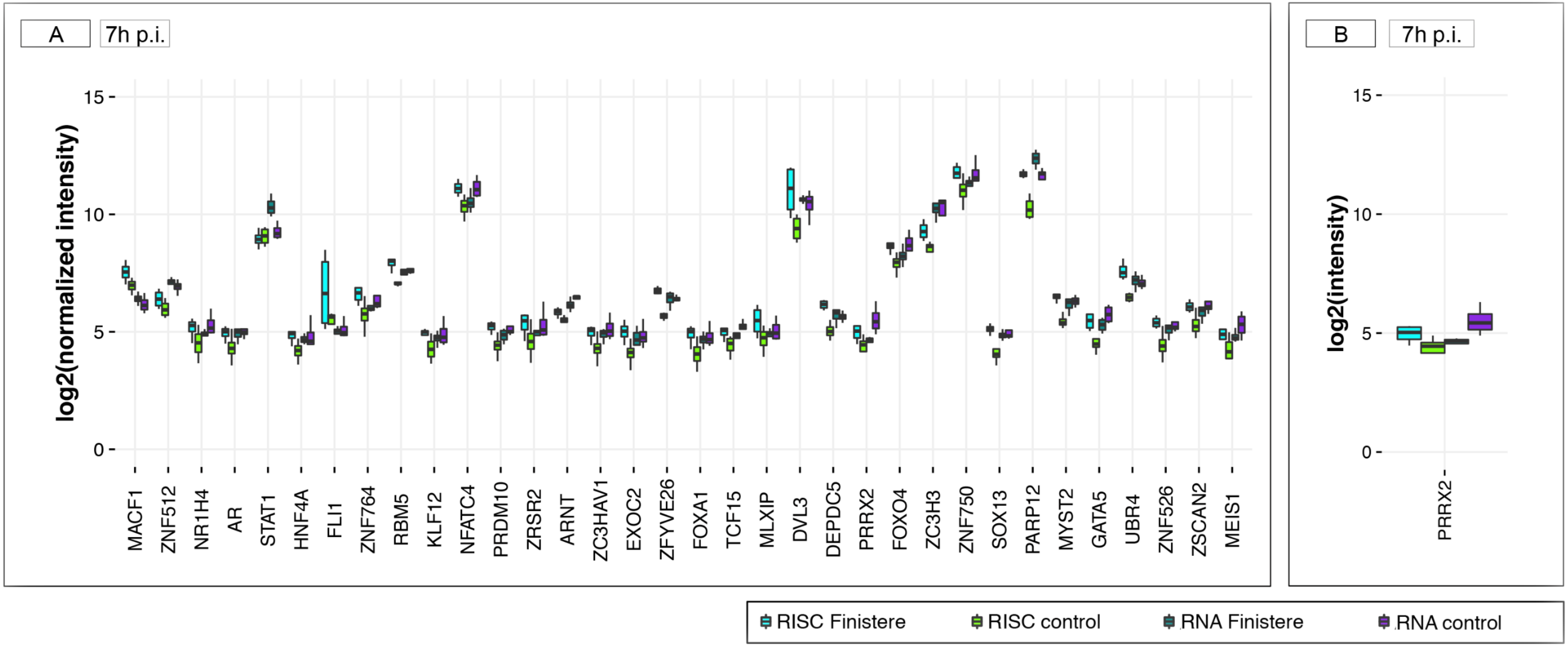

Figure S3. Transcription factors identified in the RISC compartment at 7h post-infection. (A) Expression levels of the transcription factors (TFs) identified among RISC-bound genes at 7h p.i. Both the expression in the RISC and in the whole cell is depicted. Boxes show median and interquartile range, and whiskers indicate 5th to 95th percentile. In each plot, RISC component after virus infection (cyan), RISC component control (light green), whole cell after virus infection (dark green) and control in the whole cell (violet). (B) *PRRX2* (paired-related homeobox 2) was the only TF found to be significantly represented in RISC and DEG (downregulated) in the whole cell.

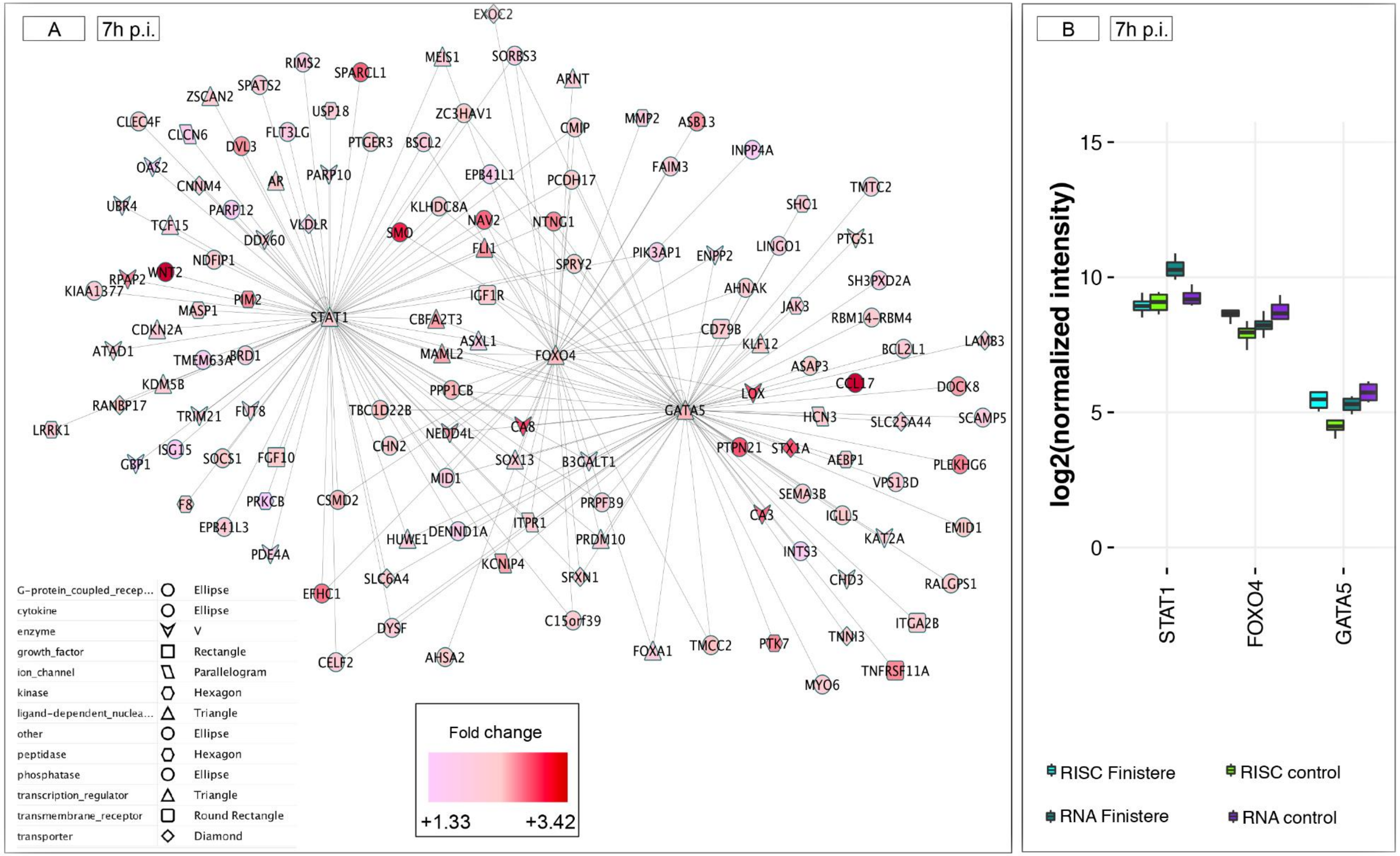

Figure S4. Relationship between enriched transcription factors (TFs) and genes in the RISC compartment at 7h p.i. (A) A schematic view of the associations between TFs and targeted genes using iRegulon. The co-association between two genes is indicated under each edge. The network is displayed graphically as nodes (genes, TFs) and edges (biological relationships). The node colour intensity indicates the FC level of the gene: higher the red intensity, higher the FC value of the enriched gene. The node shape indicates the type of gene. *STAT1* was the top potential regulator, followed by *FOXO4* and *GATA5*; (B) The boxplot graph represents the expression levels (log2) of the main candidate TFs (discovered using iRegulon) controlling RISC-bound genes in the control and infected conditions.

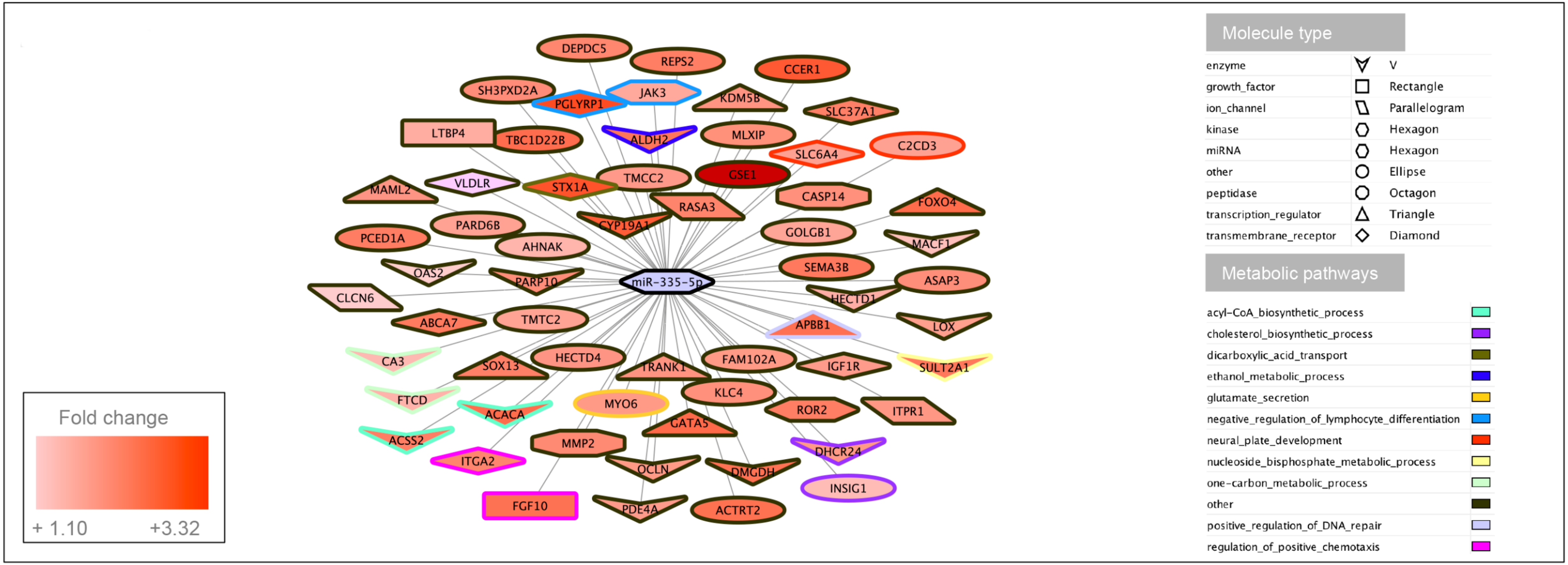

Figure S5. Regulatory network linking the miR-335-5p and their respective target genes at 7h p.i. Network representation of the 62 enriched RISC-bound genes targeted by miR-335-5p. Seven out of the 62 putative targets are transcription factors (*KDM5B*, lysine demethylase 5B; *MAML2*, mastermind like transcriptional coactivator 2; *FOXO4; APBB1*, amyloid beta precursor protein binding family B member 1; *SOX13*, SRY-box 13; *TRANK1* and *GATA5*). The network was automatically laid out using the organic layout algorithm in Cytoscape V2.7. In parallel, the list of targeted genes was fed into Cytoscape plugin ClueGo[71] to identify relevant categories of molecular functions, cellular components and biological processes. The ClueGO cutoff for the statistical assessment was FDR < 0.05. Additionally, the list of co-associated genes was uploaded to Ingenuity Pathway Analysis (IPA, version 5.5; Ingenuity Systems, USA) to define information of molecule type. Genes in network were colored based on their FC values of enrichment at 7h p.i. Gene borders in network were colored based on the ClueGO biological process that the gene participates in.

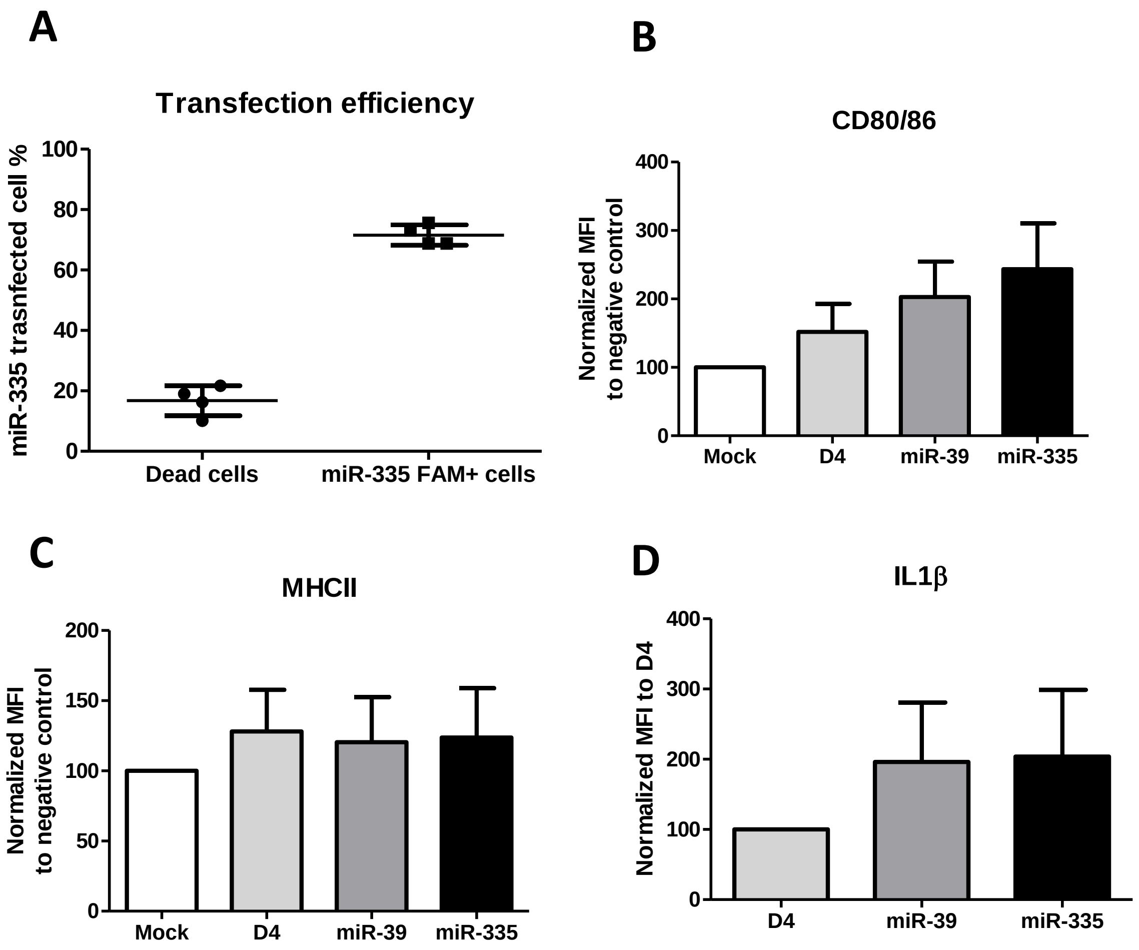

Figure S6. miR-335-5p mimics of primary PAMs. Cells were transfected with hsa-miR335-5p (miR-335), miR-335 FAM (FITC labelled) and the control cel-miR-39-3p (miR-39) at 50 nM for 30h. Data are representative of 4 independent experiments. A) The transfection efficiency was assessed by measuring the miR-335 FAM+ cells percentage by flow cytometry (FCM). Percentages of viable/dead cells were evaluated with DAPI staining. CD80 and CD86 (B) and MHCII (C) expression levels were measured by FCM while (D) IL1β production was assessed with CBA (cytometric bead array); all data are expressed as fold changes normalized vs. mean fluorescence intensities. Mock: negative control cells incubated with siRNA buffer and transfection medium alone; D4: transfection control cells incubated with siRNA buffer, DharmaFECT4 and transfection medium.

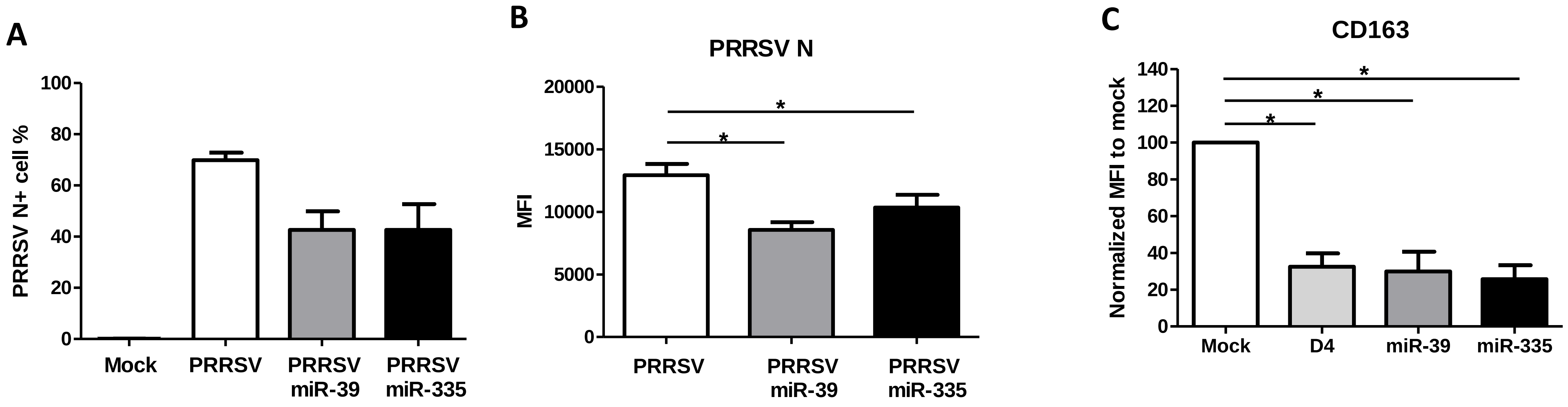

Figure S7. manipulation by miRNA mimics inhibits PRRSV replication. PAMs were transfected for miRNA mimics (see Suppl. Fig. S6) and infected with PRRSV (Finistère strain) at MOI=0.01 for 35h. PRRSV replication was compared to that of non-transfected/ infected PAMs ("PRRSV"). A) Percentage of infected cells; B) Intracellular staining of PRRSV nucleoprotein (N) expressed in absolute mean fluorescence intensity values C) CD163 receptor expression. All data were measured by FCM and normalized as fold change relative to Mock or "PRRSV". Significance was assessed by the Wilcoxon test (*P<0.05).* MFI: mean fluorescence intensity; Mock: negative control cells incubated with siRNA buffer and transfection medium alone.

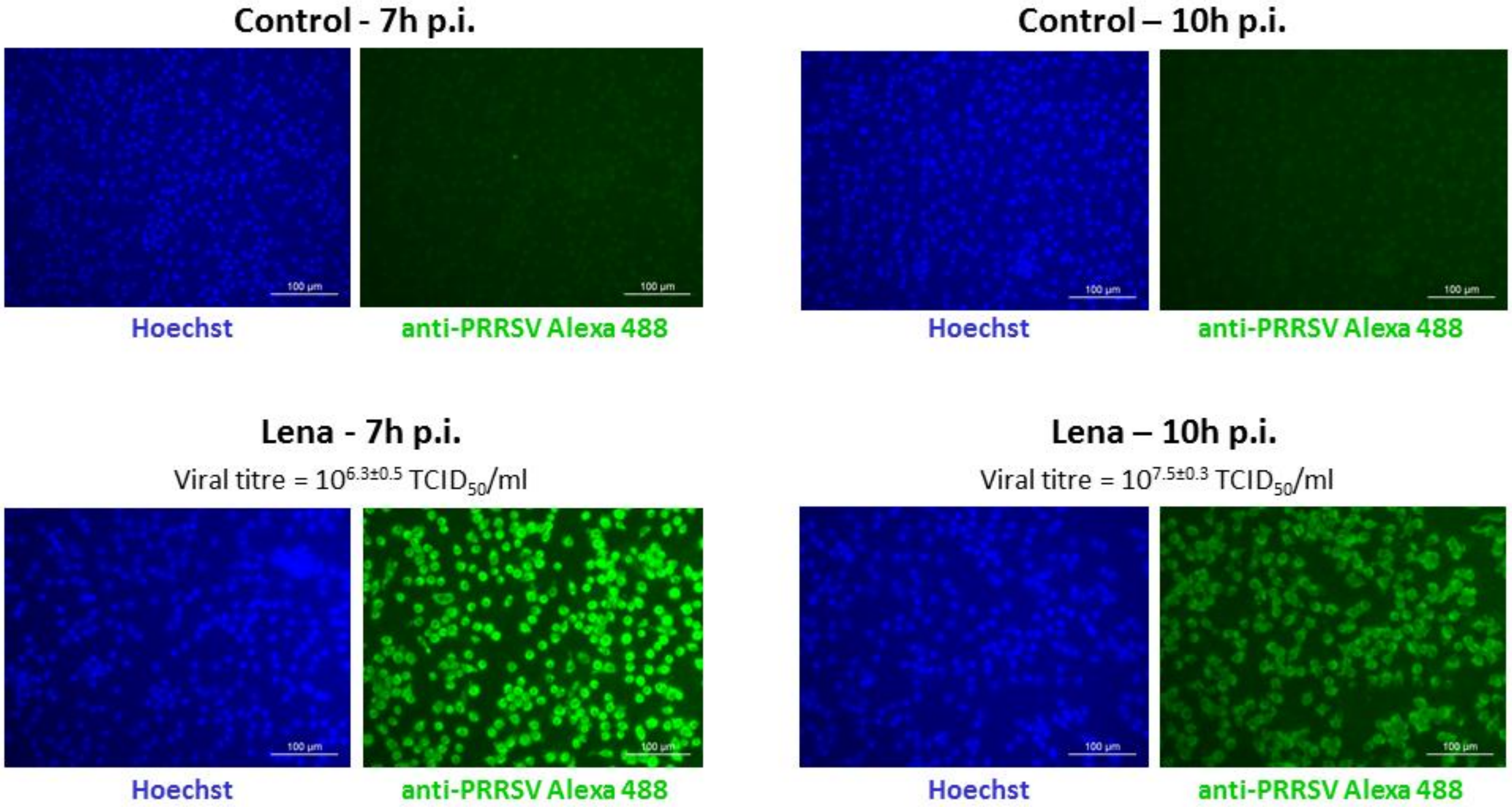

Figure S8: PRRSV immunofluorescence staining of PAMs infected with the high virulent Lena strain at 7h and 10h post-infection (at MOI= 2) and controls. PRRSV indirect staining was performed with anti-PRRSV N protein antibody and anti-IgG Alexa 488-conjugated antibody (green). The nuclei were stained with Hoechst (blue). Magnification: 200X. Images are representative of two biological replicates with three technical replicates for each experimental condition. Viral titres are means ± standard deviations of two biological replicates with three technical replicates for each experimental condition.
